# HyperSeg-DG: multi-scale hyper feature context for domain-generalized medical image segmentation

**DOI:** 10.1093/bioinformatics/btag364

**Published:** 2026-06-18

**Authors:** Md Aynul Islam, Md Youshuf Khan Rakib, Zhangjin Huang, Wang XingFu, Wenjie Du

**Affiliations:** School of Computer Science and Technology, University of Science and Technology of China, Hefei, Anhui 230027, China; School of Computer Science and Technology, Central South University, Changsha, Hunan 410083, China; School of Computer Science and Technology, University of Science and Technology of China, Hefei, Anhui 230027, China; School of Computer Science and Technology, University of Science and Technology of China, Hefei, Anhui 230027, China; School of Software Engineering, University of Science and Technology of China, Hefei, Anhui 230026, China

## Abstract

**Motivation:**

Developing segmentation models that remain reliable across diverse medical imaging domains and accurately delineate complex anatomical boundaries remains a persistent challenge for clinical deployment. Variations in imaging modalities, scanners, and acquisition settings introduce significant domain shifts, while fuzzy or overlapping tissue boundaries further complicate precise segmentation. Despite extensive research, most approaches address these challenges separately, leading to limited generalization and reduced robustness in real-world clinical scenarios.

**Results:**

To overcome these limitations, we propose HyperSeg-DG, a novel medical image segmentation approach that integrates the WMamba backbone with the Multi-Scale Hyper Feature Context Block (HFCB). The HFCB addresses foreground–background uncertainty and boundary ambiguities by capturing multi-scale feature relations and long-range contextual dependencies. This enables the model to focus on relevant pathological features while helping reduce the influence of irrelevant co-occurring ones, such as similarly sized polyps, especially in low-contrast or poorly lit environments. WMamba further improves domain generalization by processing images in localized windows and using its selective 2D scanning mechanism to learn robust, transferable features that reduce feature misalignment under domain shift. Extensive experiments across multiple medical segmentation benchmarks demonstrate that HyperSeg-DG achieves consistent 2%–3% improvements over strong baselines, confirming its effectiveness in enhancing segmentation performance and generalization across diverse, unseen domains.

**Availability and implementation:**

The code and datasets of HyperSeg-DG are available at https://github.com/Pollob001/HyperSeg-DG.

## 1 Introduction

Despite advancements in deep learning, many medical image segmentation methods still assume training and testing samples follow the same statistical distribution, an assumption that often doesn’t hold in real-world settings. Annotating large volumes of ground truth remains a challenging and expertise-heavy task (Ouyang *et al.* 2020, Wang *et al.* 2020, You *et al.* 2022). Medical images sourced from various hospitals introduce domain shifts, making generalization a significant hurdle. Medical image segmentation is crucial for clinical decision-making, treatment planning, and disease monitoring (Xun *et al.* 2022, Qureshi *et al.* 2023). Recent deep learning approaches, such as ConDSeg (Lei *et al.* 2025), TGANet (Tomar *et al.* 2022), and DFQ (Bi *et al.* 2024), have shown great promise in improving segmentation accuracy and generalization performance across diverse datasets.

In the field of medical image segmentation, significant progress has been made using deep learning, but challenges remain when it comes to domain shift and uncertainty in segmentation boundaries. Domain adaptation methods, which rely on target domain samples during training, face limitations in generalizing to unseen domains (Bian *et al.* 2021, You *et al.* 2022). These methods often fail to handle variations in feature distributions across different imaging conditions, such as contrast, illumination, and scanning techniques, resulting in feature misalignment, particularly in the early layers of deep learning models (Wang *et al.* 2020, Zhao *et al.* 2022). Additionally, the segmentation of medical images is complicated by soft boundaries between pathological and normal tissues, caused by factors like poor contrast and low lighting (Zhang *et al.* 2025, Lei and Wang 2024). The blurred boundaries, combined with significant domain shift, make it difficult for models to accurately delineate regions of interest and maintain consistent performance, hindering both domain adaptation and generalization efforts.

Conversely, domain generalization approaches aim to learn invariant representations across multiple unseen domains (Wang *et al.* 2020, Liu *et al.* 2021). However, these methods are frequently hindered by co-occurrence phenomena intrinsic to medical imaging, such as the presence of similar-sized polyps (Chen *et al.* 2019), which can mislead models into learning spurious correlations rather than pathological features. Moreover, the challenge of ambiguous boundaries persists as standard generalization techniques often fail to adequately model the uncertainty governing foreground–background transitions. Consequently, achieving reliable segmentation remains difficult in the presence of complex anatomical features, imaging inconsistencies, and the confounding effects of co-occurring patterns.

To address the challenges posed by ambiguous boundaries, foreground–background uncertainty, and co-occurring features in medical image segmentation, the HyperSeg-DG model provides distinct yet complementary solutions. In medical imaging, especially under conditions with low contrast or soft boundaries, distinguishing between pathological regions and normal tissues is complex. The Hyper Feature Context Block (HFCB) handles foreground–background uncertainty by integrating a dynamic relation module and context bridge, which work together to capture multi-scale feature relations and long-range contextual dependencies across hierarchical levels, enabling improved boundary refinement in the presence of blurry boundaries between pathological and healthy tissues. This approach is designed to reduce irrelevant co-occurring features, such as similarly sized polyps in endoscopic images, encouraging the model to focus on more meaningful pathological patterns.

Furthermore, WMamba is designed to address domain generalization by learning robust, transferable features through its selective 2D scanning mechanism. Unlike traditional models, which often struggle with feature misalignment caused by domain shifts, WMamba processes images in smaller, localized windows, enabling the model to focus on relevant spatial interactions. This localized processing enhances the model’s ability to generalize across unseen domains and handle variability in medical datasets, such as imaging inconsistencies and complex anatomical features. By combining these two techniques, our approach not only improves segmentation performance in challenging clinical conditions but also contributes to improved generalization and robustness across diverse unseen domains.

To support our workflow and achieve comparable performance with the best expert models, extensive experiments were conducted across multiple medical image segmentation benchmarks to validate the effectiveness of the proposed method. The experiments were carefully designed to assess both segmentation accuracy and domain generalization performance under varying conditions. Through these experiments, we demonstrate that our proposed method achieves consistent improvements in segmentation tasks and robust generalization across diverse datasets. The main contributions of this study are as follows:

1. We propose HyperSeg-DG, a two-stage framework for domain-generalized medical image segmentation. Stage I trains WMamba to extract hierarchical, domain-invariant features, while Stage II integrates the HFCB block with a pretrained backbone for enhanced refinement. HFCB captures multi-scale feature relations and long-range dependencies through dynamic relation modules and context bridges, enabling precise segmentation of ambiguous boundaries. By decoupling features into foreground, background, and uncertainty components, the framework reduces irrelevant co-occurring features and focuses on pathological patterns across diverse domains.
2. We propose WMamba, a hierarchical vision backbone that addresses domain generalization through localized window-based processing with four-directional selective state-space modeling. By learning spatially-adaptive, domain-invariant features within local windows rather than globally, WMamba effectively handles diverse imaging protocols and cross-domain variations. This localized approach significantly enhances generalization across heterogeneous medical datasets from different clinical settings.
3. We conduct comprehensive experiments across 10 benchmark datasets, demonstrating consistent 2–3% improvements over strong baselines. We rigorously validate domain generalization capabilities on Fundus and Prostate benchmarks, achieving substantial improvements in cross-domain stability and robustness.

## 2 Materials and methods

### 2.1 Overview

**Figure 1:**
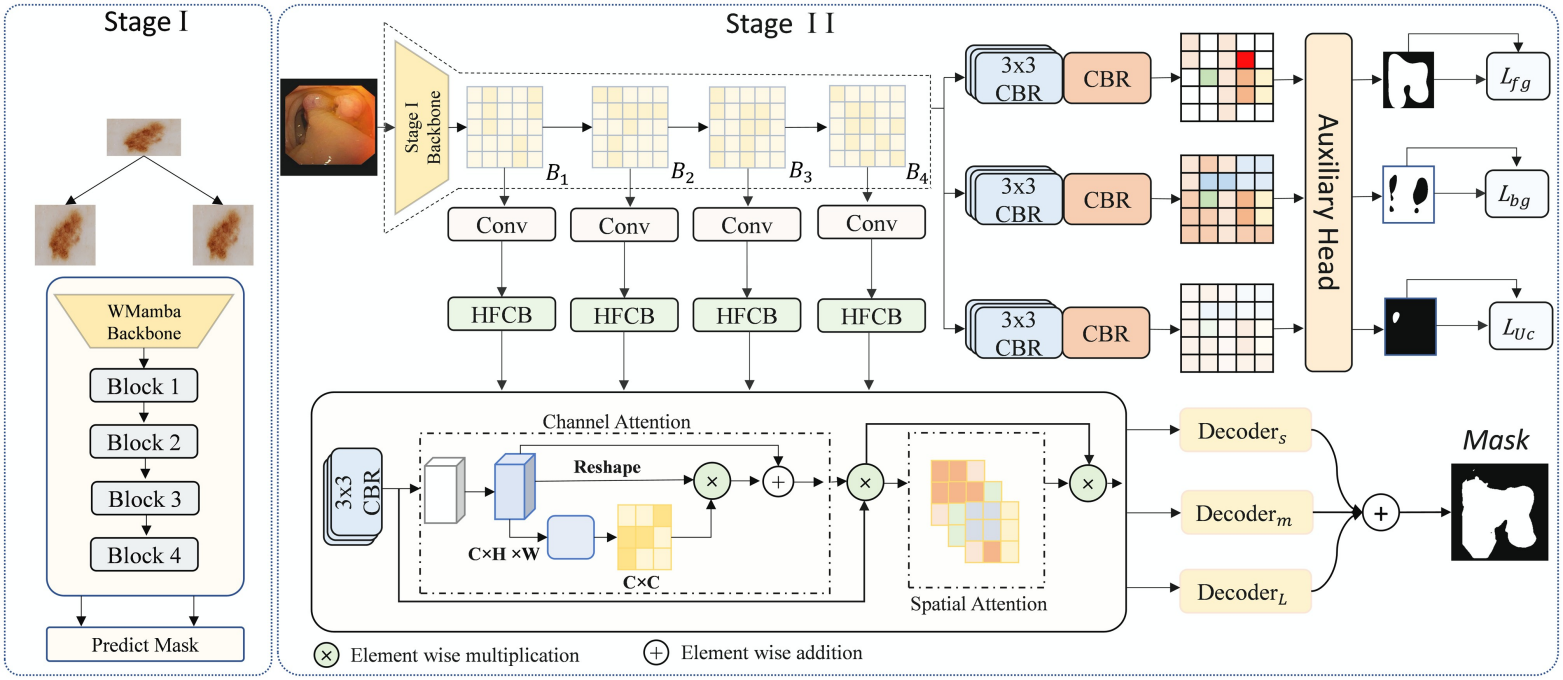
The proposed HyperSeg-DG framework for segmentation and domain generalization.

We propose HyperSeg-DG, a two-stage hierarchical framework for medical image segmentation illustrated in Fig. 1. Integrating robust visual representation learning and explicit uncertainty modeling, the framework comprises three key components: (i) Backbone Pre-training, which utilizes the WMamba architecture pre-trained on ImageNet-1K. (ii) Stage-I, an initial segmentation network employing this backbone with progressive decoding, and (iii) Stage-II, a refinement network incorporating foreground–background-uncertainty decoupling and Multi-Scale Hyper Feature Context (HFCB) blocks. Formally, given an input image $I\in R^{H\times W\times 3}$, the complete pipeline generates a refined segmentation map $M\in {\left[0,1\right]}^{H\times W}$ with explicit uncertainty quantification.

**Figure 2:**
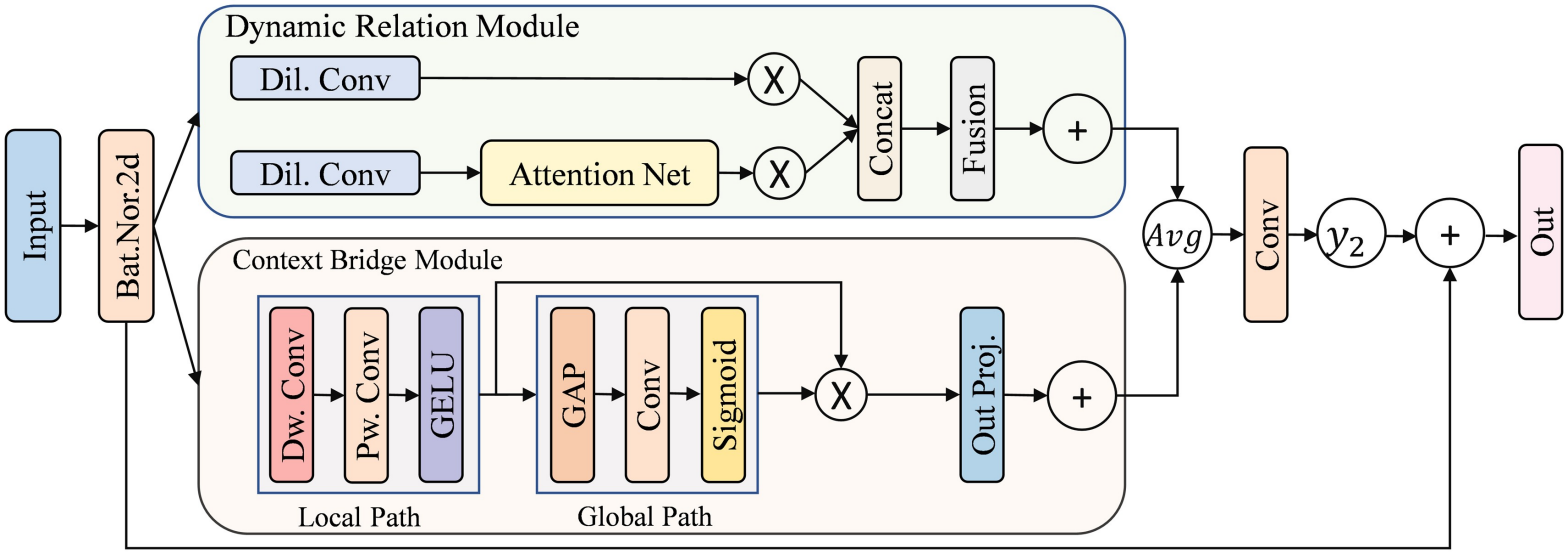
Architecture of the Multi-Scale Hyper Feature Context Block.

### 2.2 WMamba Backbone

To establish robust visual feature learning, we employ WMamba, a hierarchical backbone that synergizes the windowing strategy of Swin Transformers with the efficient long-range dependency modeling of Mamba. Distinct from VMamba (Liu *et al.* 2024a), which applies Selective Scan 2D (SS2D) globally across feature maps, or SwinMamba (Zhu *et al.* 2026), which typically integrates Mamba blocks into decoding stages, WMamba strictly enforces a window-based selective scan mechanism within the pre-trained encoder. This design localizes the linear complexity of State Space Models to partitioned windows (w×w), enhancing local feature granularity while maintaining computational efficiency. Formally, the architecture processes an input image I∈R through a hierarchical pipeline. A patch embedding layer p=4 first projects the image into a feature space $F^{\left(0\right)}\in R$, which then traverses four progressive stages to generate multi-scale representations as Equation (1),


(1) $$F^{\left(i\right)}=W^{\left(i\right)}(F^{\left(i-1\right)}),\;i\in \{1,2,3,4\}$$

where, $W^{\left(i\right)}$ denotes the WMamba layer, consisting of $i^{th}$ blocks followed by patch merging excluding the final stage. The core computational unit within each block is the window-based selective scan, designed to localize linear complexity. The feature map $X\in R^{H^{\prime }\times W^{\prime }\times C}$ is partitioned into non-overlapping windows as Equation (2),


(2) $$X_{\text{win}}=\text{Partition}(X,w)\in R^{N_{w}\times w\times w\times C}$$

where $N_{w}=\frac{H^{\prime }W^{\prime }}{w^{2}}$. The SS2D operator is then applied strictly within each window, aggregating information through four-directional scanning as Equation (3),


(3) $$Y_{\text{win}}=\sum_{k=1}^{4}{\text{Scan}}_{k}(X_{\text{win}}^{k},A_{k},B_{k},C_{k},D_{k})$$

The discrete dynamics for each direction *k*, utilizing an input-dependent time step $\Delta_{t}^{\left(k\right)}$, are governed by the state-space module equations as Equations (4) and (5),


(4) $$h_{t}^{\left(k\right)}=A_{t}^{\left(k\right)}h_{t-1}^{\left(k\right)}+B_{t}^{\left(k\right)}x_{t}^{\left(k\right)}$$

(5) $$y_{t}^{\left(k\right)}=C_{t}^{\left(k\right)}h_{t}^{\left(k\right)}+D_{t}^{\left(k\right)}x_{t}^{\left(k\right)}$$

To ensure the learned features are transferable to downstream segmentation tasks, the backbone is pre-trained on ImageNet-1K classification. The output of the final stage is processed via global average pooling (GAP) and a linear classifier to produce class probabilities $\hat{y}\in R^{1000}$. Optimization is performed using the cross-entropy loss as Equation (6). Detailed architectural specifications of WMamba and corresponding efficiency results are provided in Section S4, available as supplementary data at *Bioinformatics* online.


(6) $$L_{\text{pretrain}}=-\sum_{c=1}^{1000}y_{c}\;\text{log}(\text{softmax}({\hat{y}}_{c}))$$

### 2.3 Area definition and co-occurrence modeling

Consistency Reinforcement (Lei *et al.* 2025) ensures feature consistency across training stages. To implement this in Stage-I, we utilize the pre-trained WMamba backbone from Section 2.2 as the foundational encoder. We adapt the architecture for segmentation by discarding the classification head while retaining the hierarchical feature extractors. Consequently, given an input $I\in R^{H\times W\times 3}$, the encoder generates a multi-scale feature pyramid ${\left\{X^{\left(i\right)}\right\}}_{i=1}^{4}$ consistent with the pre-trained features $X^{\left(i\right)}\in R^{\frac{H}{2^{i+1}}\times \frac{W}{2^{i+1}}\times C_{i}}$, where the channel dimensions for the WMamba-Base variant are defined as C={128,256,512,1024}. This formulation preserves the structural integrity of learned representations while remaining resolution-agnostic.

### 2.4 Uncertainty-aware refinement network

While Stage-I provides a strong initial prediction, it often struggles with ambiguous boundaries typical in medical imaging. To address this, Stage-II introduces an explicit uncertainty modeling mechanism. We initialize the backbone with Stage-I weights to ensure representation consistency, then freeze or slowly fine-tune it while training a novel refinement branch designed to decouple and resolve semantic ambiguity.

#### 2.4.1 Uncertainty-aware feature decoupling

Standard segmentation networks implicitly force a hard decision at boundaries, leading to jagged edges. To mitigate this, we propose explicitly decoupling the feature space into three distinct semantic modes as confident foreground, background, and uncertain regions. To capture the necessary multi-scale context for this separation, we process Stage-I features ${\left\{X^{\left(i\right)}\right\}}_{i=1}^{4}$ through multi-rate dilated convolutions, yielding contextual features $D^{\left(i\right)}$. Subsequently, at the deepest semantic level, we project the representation into three parallel branches (Equation 7),


(7) $$F_{m}=\phi_{m}(X^{\left(4\right)};\Theta_{m})\in R^{8\times 8\times 128},\;m\in \{\text{fg},\text{bg},\text{uc}\}$$

where $\phi_{m}$ represents mode-specific extractors. This explicitly separates semantic content $F_{\text{fg}},F_{\text{bg}}$ and $F_{\text{uc}}$ from boundary ambiguity. To ensure these branches learn their intended roles, we enforce auxiliary supervision on each via progressive upsampling.

#### 2.4.2 Multi-scale hyper feature context

Decoupled features alone are insufficient without interaction. The network must leverage confident regions to resolve uncertain ones. The HFCB module establishes these cross-mode dependencies across scales.

We first spatially align the decoupled features to each encoder scale ℓ via iterative upsampling, denoted as $F_{m}^{\left(\ell \right)}$. To capture scale-specific structural patterns, we apply dynamic relation modeling using adaptive weights $\alpha_{m}^{\left(\ell \right)}$ as Equation (8),


(8) $$R_{m}^{\left(\ell \right)}=\sum_{j=1}^{2}\alpha_{m,j}^{\left(\ell \right)}\cdot {\text{Conv}}_{3\times 3}^{\left(d_{j}\right)}(F_{m}^{\left(\ell \right)})$$

To bridge global and local contexts, we employ a gating mechanism that enriches the features with global statistics (*G*) and local detail (*L*), fused into a bridged feature (*C*) as Equations (9–11),


(9) $$G_{m}^{\left(\ell \right)}=R_{m}^{\left(\ell \right)}⊙\sigma ({\text{Conv}}_{1\times 1}(\text{GAP}\;(R_{m}^{\left(\ell \right)})))$$

(10) $$L_{m}^{\left(\ell \right)}={\text{Conv}}_{1\times 1}(\text{GELU}({\text{DWConv}}_{3\times 3}(R_{m}^{\left(\ell \right)})))$$

(11) $$C_{m}^{\left(\ell \right)}={\text{Conv}}_{1\times 1}(G_{m}^{\left(\ell \right)}+L_{m}^{\left(\ell \right)})+R_{m}^{\left(\ell \right)}$$

Finally, to synthesize this information into a unified context, the modes interact through an adaptive fusion mechanism as Equation (12).


(12) $$H^{\left(\ell \right)}=\sum_{m\in \{\text{fg},\text{bg},\text{uc}\}}\beta_{m}^{\left(\ell \right)}\cdot C_{m}^{\left(\ell \right)}$$

where $\beta_{m}^{\left(\ell \right)}$ are learnable importance weights. This results in a rich context representation $H^{\left(\ell \right)}$ that highlights areas requiring refinement.

#### 2.4.3 Hierarchical decoding and optimization strategy

To translate the uncertainty-aware context into a refined segmentation, we employ a hierarchical integration strategy. The context $H^{\left(\ell \right)}$ is fused with the dilated spatial features $D^{\left(\ell \right)}$ and prior decoder states via residual blocks as Equation (13),


(13) $$Z^{\left(\ell \right)}={\text{Conv}}_{3\times 3}([D^{\left(\ell \right)};{\text{Up}}_{2\times 2}(Z^{\left(\ell +1\right)});H^{\left(\ell \right)}])$$

These aggregated features pass through a progressive decoder to generate scale-specific outputs $O_{\text{scale}}$, which are spatially aligned and fused to produce the final refinement $M_{\text{final}}$ as Equation (14),


(14) $$M_{\text{final}}=\sigma ({\text{Conv}}_{1\times 1}({\text{Conv}}_{3\times 3}({\text{Up}}_{4\times 4}(Z^{\left(\ell \right)}))))$$

To ensure stable convergence, we adopt a three-phase training strategy: (i) Backbone pre-training on ImageNet, (ii) Stage-I adaptation, and (iii) Stage-II refinement. Crucially, to supervise the uncertainty branch without manual labels, we generate pseudo-ground truths using Gaussian-smoothed gradients of the mask, $G_{\sigma }(|\nabla M_{\text{gt}}|)$. The total objective combines final and auxiliary losses as Equation (15),


(15) $$L_{\text{stage}2}=L_{\text{final}}+\lambda_{\text{aux}}\sum_{m\in \{\text{fg},\text{bg},\text{uc}\}}\left(L_{\text{BCE}}^{\left(m\right)}+L_{\text{Dice}}^{\left(m\right)}\right)$$

where $\lambda_{\text{aux}}=0.4$, ensuring the network prioritizes final accuracy while maintaining semantic interpretability in the decoupling branches.

### 2.5 Decoding generalized representation

We aim to learn a segmentation model $F_{\theta }:x\to y$ that generalizes effectively across domains. Let $D_{k}={\left\{(x_{n}^{\left(k\right)},y_{n}^{\left(k\right)})\right\}}_{n=1}^{N_{k}}$ represent the image-label pairs for source domains k=1,2,…,K. The target domain is defined as $D_{K+1}={\left\{x_{n}^{\left(K+1\right)}\right\}}_{n=1}^{N_{K+1}}$, containing only unlabeled images. Our objective is to learn robust representations from the source domains $D_{1\ldots K}$ that generalize to the unseen target $D_{K+1}$.

As depicted in Fig. 1, following Stage-I training, the WMamba backbone extracts hierarchical features through multiple blocks. These are denoted as $F_{1}\in R^{C_{1}\times H_{1}\times W_{1}}$, $F_{2}\in R^{C_{2}\times H_{2}\times W_{2}}$, $F_{3}\in R^{C_{3}\times H_{3}\times W_{3}}$, and $F_{4}\in R^{C_{4}\times H_{4}\times W_{4}}$, where each $F_{k}$ corresponds to features from the *k*-th WMamba block at progressively lower resolutions.

## 3 Experiments

### 3.1 Datasets

To ensure a comprehensive evaluation, we utilize a suite of ten datasets spanning diverse imaging modalities. Our experimental protocol includes eight in-distribution benchmarks for standard segmentation performance, such as BUSI (Al-Dhabyani *et al.* 2020), Kvasir-SEG (Jha *et al.* 2020), Kvasir-Sessile (Lei *et al.* 2025), CVC-ClinicDB (Bernal *et al.* 2015), GlaS (Liu *et al.* 2024b), ISIC 2016 (Gutman *et al.* 2016), ISIC 2017 (Codella *et al.* 2018), and ISIC 2018 (Codella *et al.* 2019). Furthermore, to rigorously assess the model’s generalizability to unseen domains without fine-tuning, we employ two domain generalization benchmarks DGFundus (Wang *et al.* 2020) for retinal fundus images and DGProstate (Orlando *et al.* 2020) for prostate MRI images. More detailed dataset specifications are provided in Table S2, available as supplementary data at *Bioinformatics* online.

### 3.2 Implementation details

We implement the proposed framework in PyTorch and train it using Adam with a batch size of 8. All input images were resized to 256 × 256, except for GlaS, which was kept at 512 × 512 to better preserve fine-grained structural information. A two-stage training protocol is adopted, where Stage I uses a learning rate of $1\times 10^{-4}$, and Stage II fine-tunes the encoder with $1\times 10^{-5}$ while training the refinement modules with $1\times 10^{-4}$. Backbone pretraining is performed on ImageNet-1K using 8 NVIDIA A40 GPUs, and all downstream experiments are conducted on a single NVIDIA RTX 4090 GPU. Evaluation is performed using Dice Similarity Coefficient (DSC), Average Surface Distance (ASD), mean Intersection over Union (mIoU), mean Sørensen-Dice Similarity Coefficient (mDSC), Precision, Recall, F1-score depending on the dataset and evaluation protocol. More implementation details are provided in Sections S1 and S2, available as supplementary data at *Bioinformatics* online.

## 4 Results and discussion

### 4.1 Generalized image segmentation

In real-world medical image segmentation, models are often deployed across different hospitals, scanners, imaging protocols, and patient populations, which leads to substantial domain shifts between training and testing data. We therefore evaluate HyperSeg-DG under domain-generalized settings on Fundus for optic cup segmentation and Prostate for prostate segmentation, and further assess its cross-domain robustness on ISIC for skin cancer segmentation. Specifically, the model is trained on multiple source domains and evaluated on unseen target domains without target-domain fine-tuning.

Table 1 provides a comprehensive quantitative evaluation of the proposed HyperSeg-DG against state-of-the-art domain generalization techniques for optic cup segmentation. The results unequivocally demonstrate that HyperSeg-DG achieves superior performance across all four unseen domains, exhibiting significant improvements in both region overlap and boundary accuracy. In the challenging scenarios of Domain-1 and Domain-2, where baseline methods often falter, our method improves DSC by 4.62% and 4.28% respectively over the second-best approach. More importantly, it achieves a drastic reduction in boundary error, lowering ASD by 38.55% and 38.47%. This trend of superior generalization continues in Domain-3 and Domain-4, where HyperSeg-DG attains DSC scores representing gains of 5.45% and 3.06% over the nearest competitors. The boundary refinement is particularly notable in Domain-4 where ASD is reduced by 41.37%. On average, the proposed framework sets a new benchmark. Compared to the best-performing existing method, this constitutes an overall DSC improvement of 5.00% and an ASD reduction of 37.87%, validating the efficacy of HyperSeg-DG in learning robust and domain-invariant representations for precise medical image segmentation.

**Table 1:** Performance comparison of the proposed method and existing methods on domain generalized optic cup segmentation.

Method	Domain-1		Domain-2		Domain-3		Domain-4		Average	
	DSC↑	ASD↓	DSC↑	ASD↓	DSC↑	ASD↓	DSC↑	ASD↓	DSC↑	ASD↓
Intra-domain	80.06	20.13	73.13	24.91	83.80	11.20	84.46	8.99	80.86	16.31
DeepAll	79.04	20.32	73.02	24.99	82.26	12.01	84.85	8.39	79.54	16.43
BigAug	80.37	19.50	74.73	22.64	85.39	10.07	86.47	8.32	81.74	15.13
SAML	81.03	19.31	76.61	19.31	85.40	9.99	86.06	8.86	82.78	14.62
FedDG	81.66	18.79	76.31	19.98	85.23	10.86	85.27	8.94	82.87	14.89
DoFE	81.95	18.59	78.31	16.40	85.51	10.06	86.61	8.28	83.85	13.08
RAM-DSIR	85.48	16.05	78.82	14.01	87.44	9.02	85.84	8.29	84.90	11.34
DCAC	81.43	19.20	77.72	17.15	86.80	9.14	87.68	7.12	83.41	13.15
DFQ	87.30	15.72	81.92	13.05	88.95	7.70	87.47	6.55	86.41	10.51
HyperSeg-DG	**91.33**	**9.66**	**85.43**	**8.03**	**93.80**	**4.62**	**90.36**	**3.84**	**90.73**	**6.53**

Best performance is highlighted in bold.

The results on the DGProstate dataset are shown in Table 2, where the proposed framework consistently demonstrates superior performance across all six unseen domains in terms of both DSC and ASD. In Domain-1 and Domain-2, HyperSeg-DG achieves DSC improvements of 3.38% and 2.45%, while significantly reducing ASD by 55.95% and 74.60% respectively compared to the best existing approaches. Notably, the method exhibits robust generalization in Domain-3 and Domain-4, where it improves DSC by 5.49% and 5.66% and reduces surface errors by 39.25% and 68.66%. The most substantial gain in overlap accuracy is observed in Domain-5 with a 6.64% increase in DSC and a 52.85% reduction in ASD. Furthermore, the results on Domain-6 confirm the efficacy of the approach with a 2.35% DSC improvement and a 61.02% decrease in ASD. These consistent improvements across diverse testing distributions validate the effectiveness of HyperSeg-DG in mitigating domain shifts and generating precise segmentation boundaries.

**Table 2:** Performance comparison of the proposed method and existing methods on domain generalized prostate segmentation.

Method	Domain-1		Domain-2		Domain-3		Domain-4		Domain-5		Domain-6		Average	
	DSC↑	ASD↓	DSC↑	ASD↓	DSC↑	ASD↓	DSC↑	ASD↓	DSC↑	ASD↓	DSC↑	ASD↓	DSC↑	ASD↓
Intra-domain	89.53	1.39	88.42	1.44	87.65	1.67	83.01	3.58	83.39	2.99	84.97	2.00	86.16	2.18
DeepAll	89.16	2.09	87.31	1.27	74.12	3.02	88.85	2.36	83.22	3.51	88.39	1.67	85.18	2.32
BigAug	90.68	1.80	89.52	1.00	84.86	1.86	89.04	1.59	73.24	5.94	89.10	1.16	86.07	2.23
SAML	91.00	1.26	89.26	1.12	85.76	1.87	89.60	1.21	81.60	3.29	89.91	0.96	87.86	1.62
FedDG	91.41	1.29	89.95	0.97	85.10	2.63	89.13	1.51	76.69	4.52	90.63	1.03	87.15	1.99
DoFE	89.79	1.33	87.42	1.57	84.90	2.13	88.56	1.52	86.47	1.93	87.72	1.33	87.48	1.64
RAM-DSIR	87.56	1.04	90.20	0.81	86.92	2.23	88.72	1.16	87.17	1.81	87.93	1.15	88.08	1.37
DCAC	91.76	0.98	90.51	0.89	86.30	1.77	89.13	1.53	83.39	2.46	90.56	0.85	88.61	1.41
DFQ	88.28	0.84	91.66	0.63	89.00	2.24	90.16	0.67	89.57	1.43	90.83	0.59	89.92	1.07
HyperSeg-DG	**94.86**	**0.37**	**93.91**	**0.16**	**93.89**	**1.13**	**95.26**	**0.21**	**92.21**	**0.91**	**92.76**	**0.23**	**93.82**	**0.50**

Best performance is highlighted in bold.

The results on the ISIC cross-domain benchmark are provided in Table S3, available as supplementary data at *Bioinformatics* online, where HyperSeg-DG consistently demonstrates superior performance across all three unseen domains in terms of both DSC and ASD, achieving DSC improvements of 2.50%, 2.64%, and 1.68% and ASD reductions of 21.39%, 22.11%, and 24.01% on Domain-1, Domain-2, and Domain-3, respectively, compared to the best existing method, CondSeg. These results further validate the effectiveness of HyperSeg-DG in learning robust representations and generating more accurate segmentation boundaries on unseen ISIC domains.

**Figure 3:**
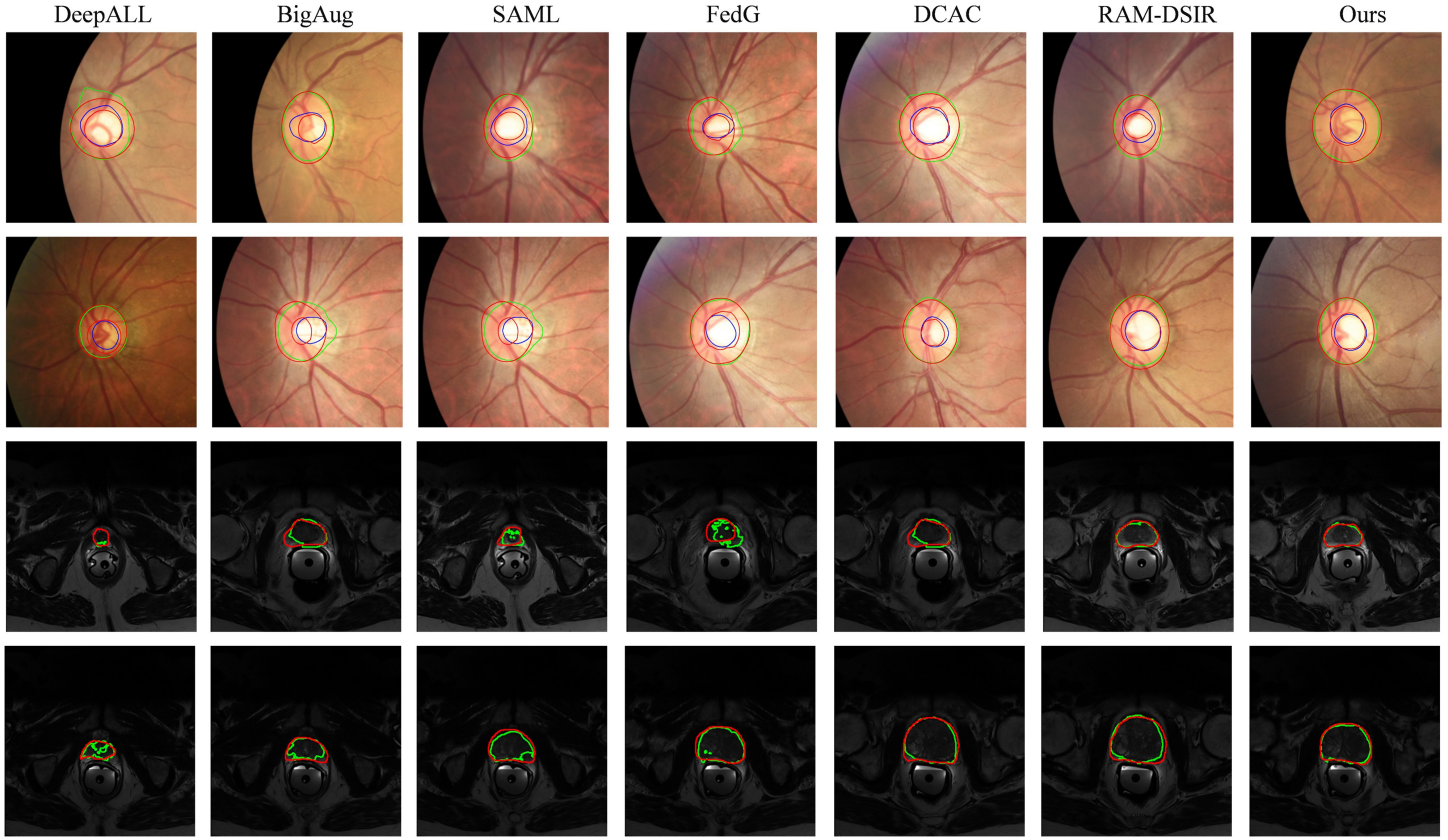
Qualitative comparison of domain generalized segmentation results between our method and state-of-the-art approaches. The first two rows display optic cup segmentation results from the Fundus benchmark, while the last two rows show prostate segmentation results from the Prostate benchmark. Green and blue overlays represent predictions from different methods, with red boundaries indicating the ground truth segmentation masks.

The qualitative comparisons in Fig. 3 further support the superiority of the proposed approach. For the Fundus benchmark, HyperSeg-DG produces segmentation contours that more closely match the ground-truth optic cup boundaries, particularly in challenging cases with ambiguous edges and low contrast. For the Prostate benchmark, our method preserves the anatomical structure more faithfully and avoids the boundary irregularities and leakage into surrounding tissues that are observed in competing approaches. These visual results are consistent with the quantitative findings and further demonstrate the strong cross-domain robustness of HyperSeg-DG.

### 4.2 In-distribution segmentation

We further evaluate HyperSeg-DG under standard in-distribution settings on multiple medical image segmentation benchmarks, including BUSI, CVC, ISIC16, Kvasir-SEG, Kvasir-Sessile, and GlaS. These experiments assess whether the proposed framework can maintain strong segmentation accuracy when training and testing data follow the same underlying distribution.

**Figure 4:**
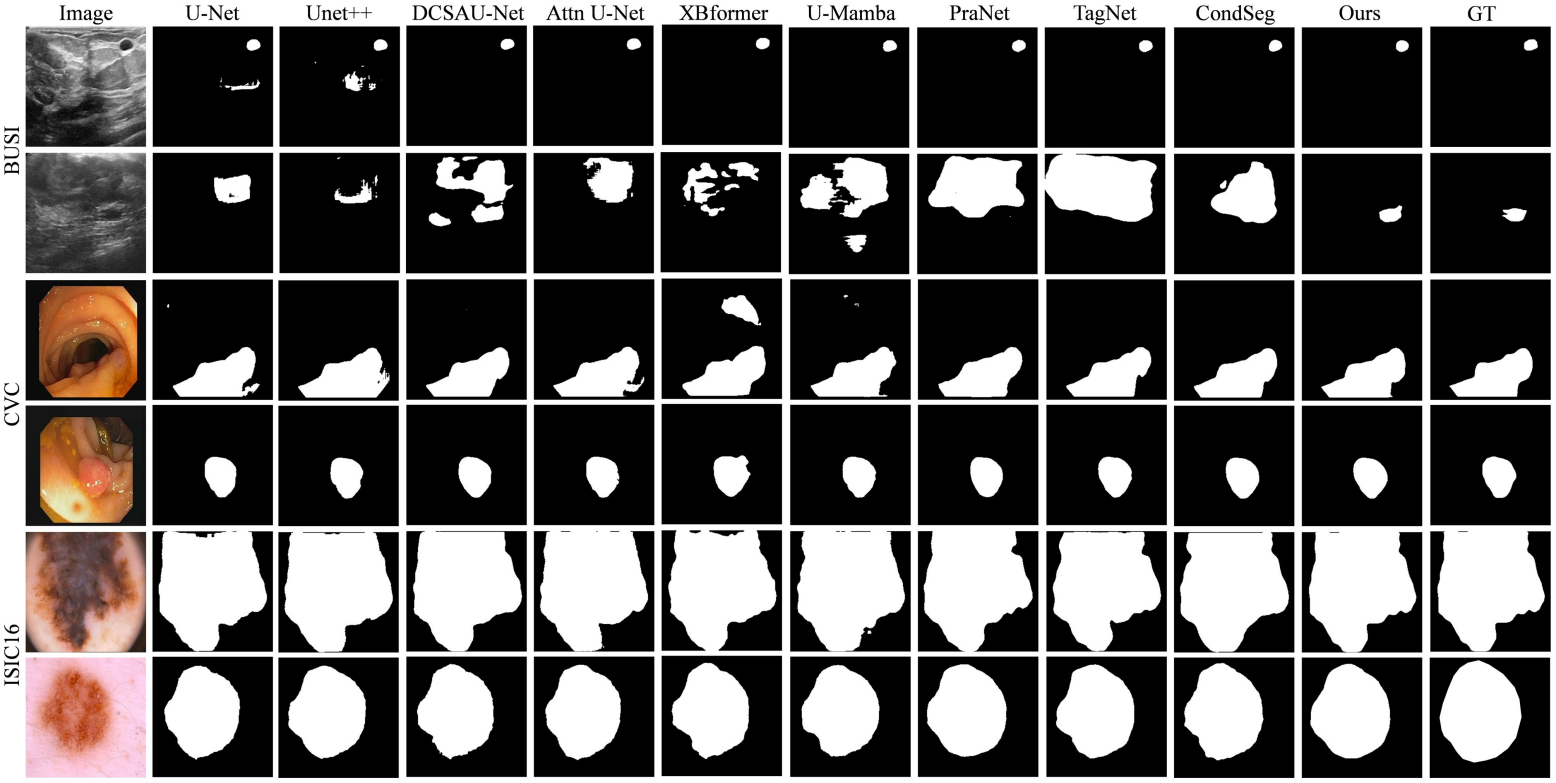
Visualization of segmentation results from state-of-the-art methods on the BUSI, CVC, ISIC-16 datasets with different modalities.

Figure 4 presents a visual comparison of segmentation results across different methods on the multimodal dataset imaging modalities as BUSI, CVC, and ISIC 2016. Our proposed method demonstrates superior boundary delineation and anatomical structure preservation compared to existing approaches. In breast ultrasound images, our method accurately captures lesion boundaries while minimizing false positives in heterogeneous tissue regions. For colonoscopy images, the method effectively segments polyps despite challenging lighting conditions and specular reflections. In dermoscopy images, precise lesion segmentation is achieved even with irregular borders and hair occlusions. Extended qualitative results for Kvasir-SEG, Kvasir-Sessile, GLAS, and ISIC 2017/2018 are detailed in Figs S2 and S3, available as supplementary data at *Bioinformatics* online.

The qualitative comparison in Fig. S1, available as supplementary data at *Bioinformatics* online, demonstrates our method’s superior segmentation on Kvasir and CVC datasets. The first two columns show input images and ground truth. The third column presents single segmentation with Grad-CAM, while the fourth shows co-occurrence segmentation with Grad-CAM. The final column compares attention heatmaps. Our method achieves more accurate, focused segmentation, with sharper and more precisely localized Grad-CAM and heatmap regions, reflecting enhanced attention to relevant areas and demonstrating clear performance gains over co-occurrence-based approaches.

Table S4, available as supplementary data at *Bioinformatics* online, presents the performance comparison of various segmentation methods across three datasets such as Kvasir-Sessile, Kvasir-SEG, and GlaS. Our approach consistently outperforms all competing methods across all evaluated metrics, including mIoU, mDSC, Recall, and Precision. Specifically, on the Kvasir-Sessile dataset, our method demonstrates improvements of 1.5% in mIoU, 3.2% in mDSC, 4.2% in Recall, and 5.4% in Precision compared to the best competitor, ConDSeg. For the Kvasir-SEG dataset, we achieve enhancements of 1.5% in mIoU, 3.0% in mDSC, 3.9% in Recall, and 1.2% in Precision. On the GlaS dataset, our method outperforms ConDSeg by 3.0% in mIoU, 0.7% in mDSC, 1.3% in Recall, and 1.6% in Precision. These results show that HyperSeg-DG remains strong not only under cross-domain evaluation but also on standard in-distribution segmentation benchmarks.

### 4.3 Ablation study

As shown in Table 3, our full model consistently outperforms all ablation variants across all domains. The variant without pretrained weights degrades performance by 3.79% in DSC and 28.6% in ASD on average, demonstrating the importance of pretraining. Replacing WMamba with Swin Transformer leads to a drop of 3.42% in DSC and 32.1% in ASD, validating the superiority of our proposed WMamba backbone. Removing the HFCB module reduces DSC by 2.23% and increases ASD by 25.1%. Excluding the Uncertainty Block results in a decline of 1.26% in DSC and 18.9% in ASD. Training without Stage-1 causes a decrease of 1.78% in DSC and 20.8% in ASD, confirming the necessity of two-stage training. The largest improvement is observed in Domain 3, where Ours achieves 93.80% DSC compared to 87.51% for the variant without pretrained weights, an improvement of 6.29%.

**Table 3:** Ablation evaluation of different components of HyperSeg-DG on the DGFundus dataset, where w/o pr is without pretrained weights, w/o hfcb is without HFCB, w/o ub is without Uncertainty Block, w/o wm replaces WMamba with Swin-T, and w/o s1 omits Stage-1 training.

Method	Domain 1		Domain 2		Domain 3		Domain 4	
	DSC↑	ASD↓	DSC↑	ASD↓	DSC↑	ASD↓	DSC↑	ASD↓
w/o pr	86.95	15.49	79.31	13.47	87.51	10.06	86.61	8.28
w/o hfcb	88.55	12.14	83.11	9.93	91.24	6.56	89.05	4.62
w/o ub	90.20	10.06	84.71	8.89	92.13	5.27	89.84	4.43
w/o wm	87.72	13.37	81.21	11.36	90.65	7.73	87.97	6.38
w/o s1	89.31	11.21	84.33	9.28	92.09	6.18	88.36	5.04
Ours	**91.33**	**9.66**	**85.43**	**8.03**	**93.80**	**4.62**	**90.36**	**3.84**

Best performance is highlighted in bold.

Table 4 demonstrates that while Stage II yields marginal gains over Stage I with improvements of 1.32% in mIoU and 1.82% in mDSC, the integration of both stages substantially enhances performance across all metrics. The joint strategy outperforms the Stage I baseline by 5.76% in mIoU and 4.77% in mDSC while achieving further gains of 4.38% and 2.90% over Stage II. The most significant improvement occurs in Recall, where the combined method surpasses Stage I by 5.92% and Stage II by 4.98%, which indicates a strong reduction in false negatives. Although Precision gains are smaller at 2.22% and 1.54% respectively, the overall results confirm that the two stages are highly complementary and necessary for robust segmentation.

**Table 4:** Ablation evaluation results of training strategies on GlaS dataset.

Training strategy		Metrics			
Stage1	Stage2	mIoU	mDSC	Rec.	Prec.
√		83.3	88.1	89.5	90.1
	√	84.4	89.7	90.3	90.7
√	√	**88.1**	**92.3**	**94.8**	**92.1**

Best performance is highlighted in bold.

We conduct an ablation study comparing it against the baseline architecture on the GlaS dataset. The quantitative results, summarized in Table 5, demonstrate that integrating the HFCB leads to a consistent performance boost across all evaluated metrics. Specifically, the mIoU increases from 84.1 to 88.1, representing a relative improvement of 4.76%. Similarly, the mDSC improves from 90.4 to 92.3. The enhancements in recall and precision rise by 2.60% and 2.45%, respectively.

**Table 5:** Ablation evaluation of the HFCB block on the GlaS dataset.

Modules		Metrics			
Baseline	HFCB	mIoU	mDSC	Rec.	Prec.
√		84.1	90.4	92.4	89.9
√	√	**88.1**	**92.3**	**94.8**	**92.1**

Best performance is highlighted in bold.

As shown in Table 6, WMamba consistently outperforms all compared backbone models across all metrics. Compared to Swin-T, the previous best backbone, WMamba improves mIoU by 3.13%, mDSC by 1.42%, Recall by 2.95%, and Precision by 3.47%, achieving an average improvement of 2.74% across all four metrics. Compared to VGG-19, the weakest backbone, WMamba shows substantial gains of 8.1% in mIoU, 5.0% in mDSC, 5.0% in Recall, and 4.7% in Precision. The consistent improvements over ResNet variants and PVT demonstrate the effectiveness of our proposed WMamba backbone for medical image segmentation.

**Table 6:** Ablation evaluation of backbone models on the ISIC-17 dataset.

Backbone models	mIoU	mDSC	Rec.	Prec.
VGG-19	80.8	88.1	89.1	90.7
ResNet-18	82.2	88.9	89.4	91.5
ResNet-34	83.7	89.7	90.8	92.6
ResNet-50	85.5	90.6	92.1	93.2
PVT	85.9	90.9	91.4	91.9
Swin-T	86.2	91.8	91.4	92.2
WMamba	**88.9**	**93.1**	**94.1**	**95.4**

Best performance is highlighted in bold.

## 5 Conclusions

In our paper, we proposed a novel approach for medical image segmentation that integrates the WMamba backbone with the Multi-Scale HFCB, addressing key challenges like foreground–background uncertainty, ambiguous boundaries, and domain generalization. Our method improves segmentation accuracy by capturing multi-scale features and long-range contextual dependencies, while effectively distinguishing foreground from background and handling uncertainty regions. Although the current focus is on 2D medical images, future work will extend this approach to 3D segmentation. By leveraging volumetric feature extraction and domain adaptation techniques, we aim to improve generalization across diverse 3D medical imaging datasets, such as CT and MRI scans, ensuring more robust and accurate segmentation in complex 3D structures and ultimately enhancing the model’s clinical applicability across various domains.

## Supplementary Material

btag364_Supplementary_Data

## Data Availability

The code and datasets of HyperSeg-DG are available at https://github.com/Pollob001/HyperSeg-DG.
