# Supplementary material for: HyperSeg-DG: multi-scale hyper feature context for domain-generalized medical image segmentation
Source: Bioinformatics. 2026 Jun 18;42(6):btag364. doi: 10.1093/bioinformatics/btag364 (PMC13282076; doi:10.1093/bioinformatics/btag364)
Supplement: btag364_Supplementary_Data [file btag364_supplementary_data.pdf]

Supplementary Materials for **HyperSeg-DG: Multi-Scale Hyper Feature Context for Domain Generalized Medical image Segmentation**

Md Aynul Islam[1,‡], MD Youshuf Khan Rakib[2,‡], Zhangjin Huang [1], Wang XingFu[1], and Wenjie Du[3,*]

[1]School of Computer Science and Technology, University of Science and Technology of China, Hefei 230027, Anhui, China
[2]School of Computer Science and Technology, Central South University, Changsha 410083, Hunan, China
[3]School of Software Engineering, University of Science and Technology of China, Hefei 230026, Anhui, China
[‡]Equal contribution.
[*]Corresponding Author: duwenjie@ustc.edu.cn

Motivation: Developing segmentation models that remain reliable across diverse medical imaging domains and accurately delineate complex anatomical boundaries remains a persistent challenge for clinical deployment. Variations in imaging modalities, scanners, and acquisition settings introduce significant domain shifts, while fuzzy or overlapping tissue boundaries further complicate precise segmentation. Despite extensive research, most approaches address these challenges separately, leading to limited generalization and reduced robustness in real-world clinical scenarios.
Results: To overcome these limitations, we propose HyperSeg-DG, a novel medical image segmentation approach that integrates the WMamba backbone with the Multi-Scale Hyper Feature Context Block (HFCB). The HFCB addresses foreground-background uncertainty and boundary ambiguities by capturing multi-scale feature relations and long-range contextual dependencies. This enables the model to focus on relevant pathological features while helping reduce the influence of irrelevant co-occurring ones, such as similarly sized polyps, especially in low-contrast or poorly lit environments. WMamba further improves domain generalization by processing images in localized windows and using its selective 2D scanning mechanism to learn robust, transferable features that reduce feature misalignment under domain shift. Extensive experiments across multiple medical segmentation benchmarks demonstrate that HyperSeg-DG achieves consistent 2–3% improvements over strong baselines, confirming its effectiveness in enhancing segmentation performance and generalization across diverse, unseen domains.
Availability: The code and datasets of HyperSeg-DG are available at `https://github.com/Pollob001/HyperSeg-DG`
.
Contact: Wenjie Du (duwenjie@ustc.edu.cn)

keywords: Domain generalization, multi-scale feature fusion, state-space models, uncertainty estimation

This pdf file includes:

- Supplementary Methods and Materials
- Supplementary Results
- Supplementary Tables S1–S5
- Supplementary Figures S1–S14

S1 Experimental Setup

Backbone pre-training utilizes ImageNet-1K (?) on 8 NVIDIA A40 GPUs. Downstream experiments employ a single RTX 4090, optimizing via Adam (Kingma and Ba, 2017) batch size 8 with standard augmentations. A two-stage training strategy is adopted as Stage-I uses a learning rate of 1×10^{-4}, while Stage-II fine-tunes the encoder at 1×10^{-5} and trains refinement modules at 1×10^{-4}.

Table S1: Hyperparameter settings for HyperSeg-DG.

**Parameter**	**Stage 1**	**Stage 2**	**Generalization**
Image Size	256^2	256^2	256^2 (test: $800^2 \rightarrow 256^2$)
Batch Size	8	8	8
Learning Rate	1×10^{-4}	1×10^{-4}	1×10^{-3}
Epochs	200	600	400 (Fundus), 200 (Prostate)
Early Stopping	100	100	100
Optimizer	Adam	Adam	Adam
Learning Rate Decay	ReduceLROnPlateau	ReduceLROnPlateau	Polynomial rule

S2 Evaluation Metrics

To assess segmentation performance, we adopt a comprehensive set of metrics covering region overlap, boundary accuracy, and detection quality. Each metric captures a distinct aspect of segmentation fidelity, and together they provide a thorough evaluation framework suitable for medical image analysis.

The Dice Similarity Coefficient measures volumetric overlap between the predicted mask and ground truth, defined in Eq. 1.

$$\text{DSC} = \frac{2 \times |P \cap G|}{|P| + |G|} \tag{1}$$

where P and G denote the predicted and ground truth segmentations, respectively. DSC ranges from 0 to 1, where a value of 1 indicates perfect overlap and 0 indicates no overlap. It is widely adopted in medical image segmentation due to its sensitivity to both over- and under-segmentation.

The mean Dice Similarity Coefficient extends this to multi-class settings by averaging over N classes, as given in Eq. 2.

$$\text{mDSC} = \frac{1}{N} \sum_{i=1}^{N} \frac{2 \times |P_i \cap G_i|}{|P_i| + |G_i|} \tag{2}$$

By averaging across all classes, mDSC provides a balanced measure of segmentation quality that accounts for class imbalance, which is common in clinical datasets where lesion regions are significantly smaller than background regions.

The mean Intersection over Union, also known as the Jaccard Index, captures the ratio of overlap to total coverage across all classes, as defined in Eq. 3.

$$\text{mIoU} = \frac{1}{N} \sum_{i=1}^{N} \frac{|P_i \cap G_i|}{|P_i \cup G_i|} \tag{3}$$

Unlike DSC, mIoU penalizes false positives and false negatives more aggressively, making it a stricter measure of segmentation accuracy. It is particularly informative when evaluating models on datasets with significant structural variation across classes.

Precision and Recall are further employed to characterize false positive and false negative rates, as given in Eq. 4 and Eq. 5.

$$\text{Precision} = \frac{\text{TP}}{\text{TP} + \text{FP}} \tag{4}$$

$$\text{Recall} = \frac{\text{TP}}{\text{TP} + \text{FN}} \tag{5}$$

where TP, FP, and FN refer to true positives, false positives, and false negatives, respectively. High Precision indicates that the model produces few spurious detections, while high Recall reflects the model ability to identify all relevant regions. In clinical applications, a balance between the two is essential, as both missed detections and false alarms carry diagnostic consequences.

For boundary-level evaluation, the Average Symmetric Surface Distance quantifies the mean geometric deviation between predicted and ground truth contours, as defined in Eq. 6.

$$\mathrm{ASD} = \frac{1}{|S(P)| + |S(G)|}\left(\sum_{x\in S(P)} d(x, S(G)) + \sum_{y\in S(G)} d(y, S(P))\right) \tag{6}$$

where $S(P)$ and $S(G)$ are the boundary point sets of the predicted and ground truth masks, and d is the Euclidean distance. The symmetric formulation ensures that deviations are measured in both directions, from prediction to ground truth and from ground truth to prediction, yielding a balanced assessment of contour fidelity. Lower ASD values reflect tighter boundary alignment, which is particularly critical in clinical segmentation tasks where precise delineation of anatomical structures directly influences downstream diagnosis and treatment planning. Together, these metrics form a robust and complementary evaluation protocol that assesses segmentation performance from multiple perspectives, ensuring that both region-level accuracy and boundary-level precision are thoroughly examined.

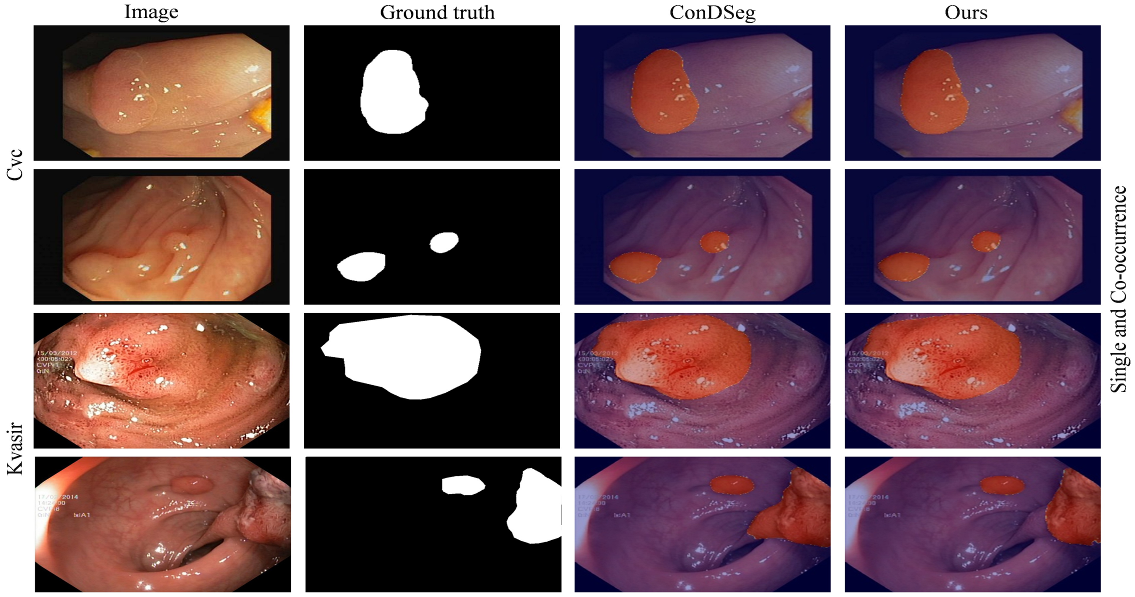

Figure S1: Qualitative comparison of segmentation results.

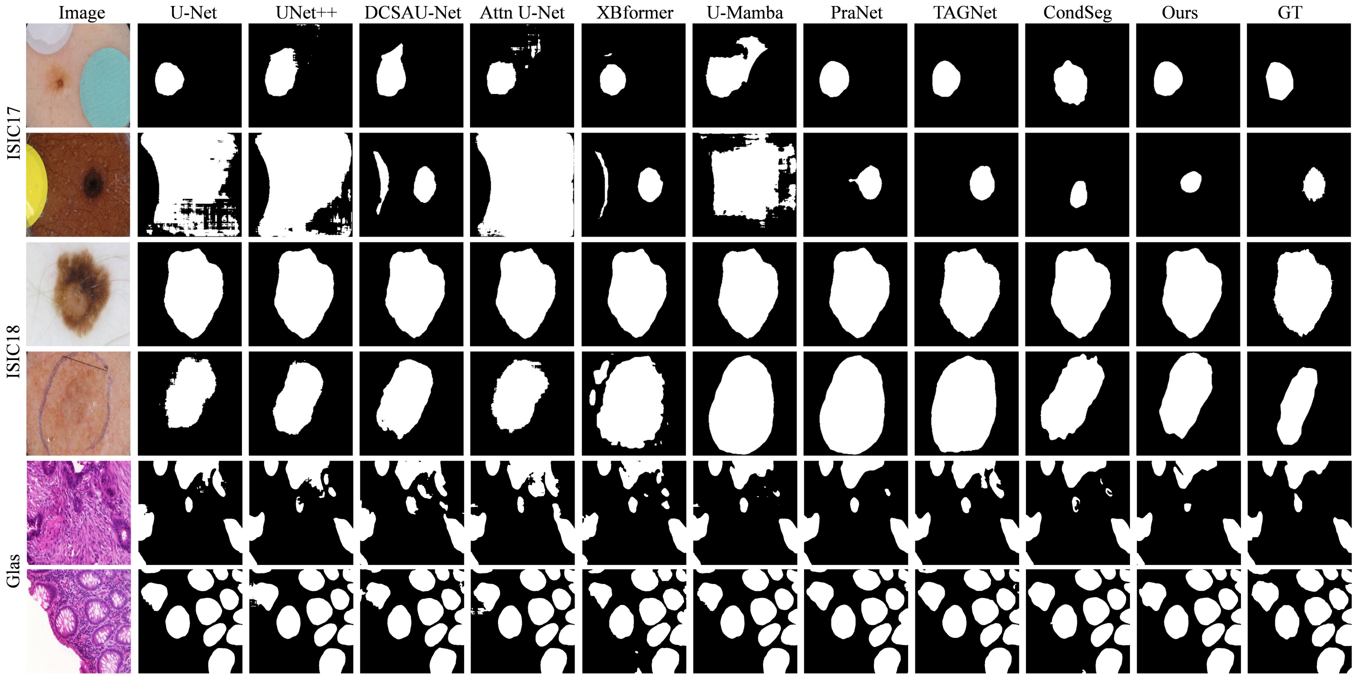

Figure S2: Segmentation performance on GLAS histology images and ISIC 2017/2018 dermoscopy datasets. The proposed method preserves morphological consistency in complex glandular structures and accurately segments lesions with irregular boundaries.

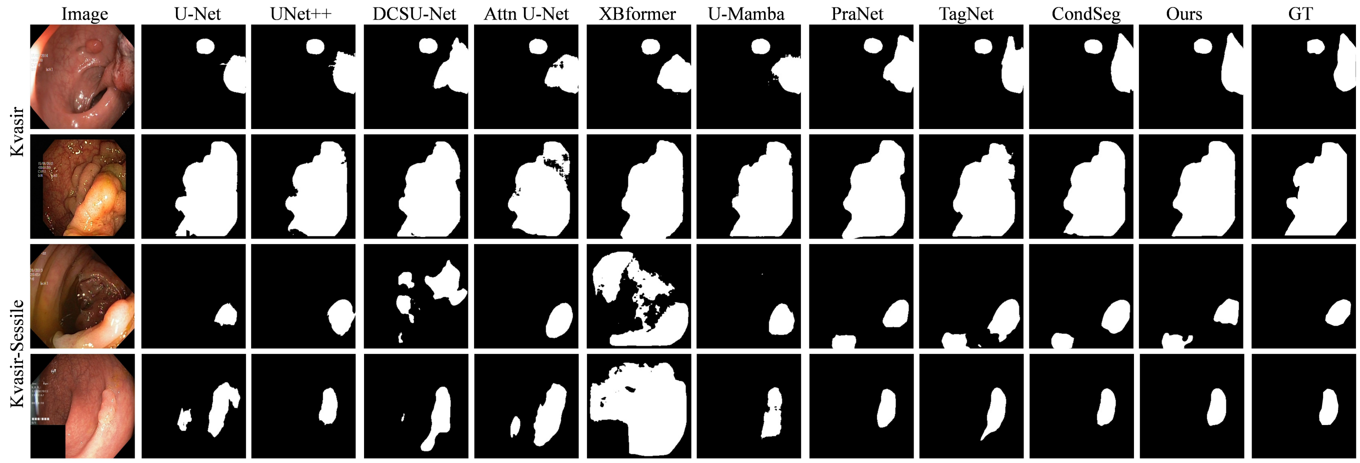

Figure S3: Segmentation results on Kvasir-SEG and Kvasir-Sessile polyp datasets, demonstrating robust performance under challenging endoscopic conditions.

Table S2: Segmentation and generalization benchmark datasets used in our study.

**Benchmark/Dataset**	**Modality**	**Organ**	**Target**	**Images/Cases**
		**Segmentation**		
BUSI	Ultrasound	Breast	Lesion	647
Kvasir-SEG	Endoscope	Colon	Polyp	1000
Kvasir-Sessile	Endoscope	Colon	Polyp	196
GlaS	(WSI)	Colorectum	Gland	165
ISIC-2016	Dermoscope	Skin	Skin Lesion	1279
ISIC-2017	Dermoscope	Skin	Skin Lesion	2750
ISIC-2018	Dermoscope	Skin	Skin Lesion	2594
CVC	Images	Colon	Polyp	612
		**Generalization**		
DGFundus	Fundus	Eye	Optic Cup/Disc	800
Domain-1	Fundus	Eye	Optic Cup/Disc	-
Domain-2	Fundus	Eye	Optic Cup/Disc	-
Domain-3	Fundus	Eye	Optic Cup/Disc	-
Domain-4	Fundus	Eye	Optic Cup/Disc	-
DGProstate	T2-weighted MRI	Prostate	Cancer	116
Domain-1	T2-weighted MRI	Prostate	Cancer	-
Domain-2	T2-weighted MRI	Prostate	Cancer	-
Domain-3	T2-weighted MRI	Prostate	Cancer	-
Domain-4	T2-weighted MRI	Prostate	Cancer	-
Domain-5	T2-weighted MRI	Prostate	Cancer	-
Domain-6	T2-weighted MRI	Prostate	Cancer	-

Table S3: Performance of different methods across ISIC datasets for generalization tasks. Best performance is highlighted in bold.

Method	Domain-1		Domain-2		Domain-3		Average	
	DSC↑	ASD↓	DSC↑	ASD↓	DSC↑	ASD↓	DSC↑	ASD↓
RAM-DSIR	83.41	18.09	81.89	15.26	87.74	13.04	84.35	15.46
Condseg	86.71	14.21	85.92	11.85	88.78	9.58	87.14	11.88
**HyperSeg-DG**	**89.21**	**11.17**	**88.56**	**9.23**	**90.46**	**7.28**	**89.41**	**9.23**

Table S4: Comparison with other methods on the Kvasir-Sessile, Kvasir-SEG and GlaS datasets.Best performance is highlighted in bold.

Methods	Kvasir-Sessile				Kvasir-SEG				GlaS			
	mIoU	mDSC	Rec.	Prec.	mIoU	mDSC	Rec.	Prec.	mIoU	mDSC	Rec.	Prec.
U-Net	23.1	33.8	45.1	46.6	65.5	75.8	83.6	77.6	75.8	85.5	90.3	82.8
U-Net++	38.4	50.2	62.5	51.8	67.9	77.2	86.5	77.6	77.6	86.9	89.6	85.5
AttnU-Net	27.5	38.9	59.0	44.4	67.6	77.4	83.9	79.9	76.6	85.9	91.8	82.2
PraNet	66.7	77.4	80.7	82.4	83.0	89.4	90.6	91.3	71.8	83.0	90.9	78.0
TGANet	74.4	82.0	79.3	85.9	83.3	89.8	91.3	91.2	71.8	84.7	86.9	80.2
DCSAU-Net	72.6	81.1	65.6	62.9	83.5	88.9	89.5	89.5	77.6	86.5	93.0	82.5
XBFormer	73.6	81.1	87.2	76.3	83.8	88.9	89.8	87.2	73.7	84.3	84.0	85.7
CASF-Net	60.5	72.4	78.0	74.8	81.7	88.7	89.2	88.2	78.4	87.2	91.3	85.9
DTAN	76.4	84.2	84.2	85.9	84.1	90.4	91.6	92.0	78.5	87.9	85.8	90.2
ConDSeg	81.2	89.1	90.1	90.0	84.6	90.5	92.3	91.7	85.1	91.6	93.5	90.5
**HyperSeg-DG**	**82.7**	**92.3**	**94.3**	**95.4**	**86.1**	**93.5**	**96.2**	**92.9**	**88.1**	**92.3**	**94.8**	**92.1**

S3 t-SNE Visualization of Learned Feature Embeddings

Figure S4 presents the t-SNE visualization of learned feature embeddings on the Fundus dataset, comparing three different settings: (left) baseline domain generalization, (middle) decoupled representation learning, and (right) the auxiliary head. The t-SNE plots visually represent the clustering of feature embeddings, where more compact and well-separated clusters indicate improved discriminative power. The results demonstrate that the auxiliary head significantly enhances the feature embeddings, leading to better-defined and more distinct clusters compared to both the baseline and decoupled representation learning settings. This improvement suggests that the auxiliary head contributes to more effective domain-invariant feature learning, ultimately enhancing model generalization across domains.

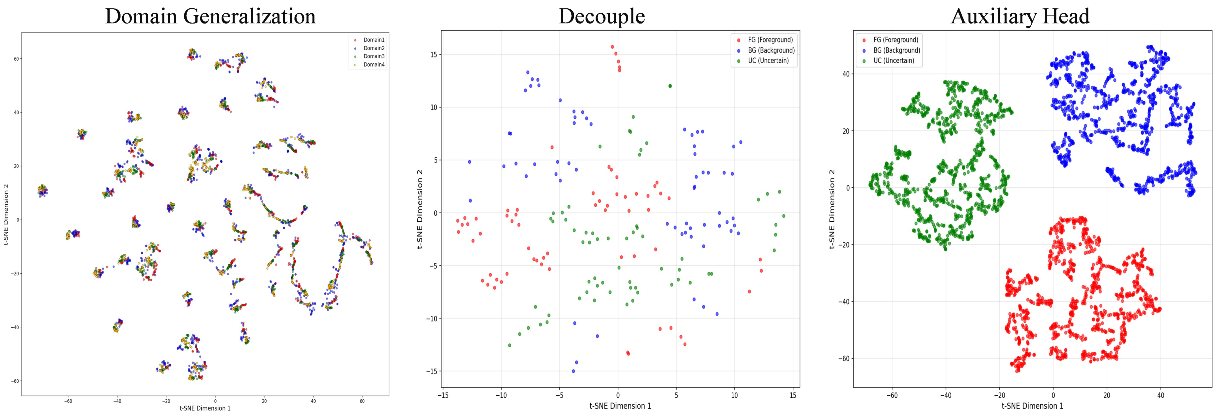

Figure S4: t-SNE visualization of learned feature embeddings on the fundus dataset under three settings: (left) baseline domain generalization, (middle) decoupled representation learning, and (right) auxiliary head. The auxiliary head yields more compact and better-separated clusters, indicating improved discriminative representation across domains.

S4 Backbone WMamba

S4.1 Architecture of WMamba

Figure S5(a) illustrates the proposed WMamba backbone, which synthesizes the hierarchical multi-scale structure of Swin Transformers with the linear complexity of State Space Models. By replacing computationally intensive self-attention with the parameter-efficient Selective Scan 2D (SS2D) operator, the architecture effectively captures local semantic granularity within windowed regions. This design advantage is quantitatively validated in Figure S5(b) and (c), where WMamba demonstrates a superior trade-off between segmentation accuracy and computational efficiency (FLOPs and inference throughput) compared to existing state-of-the-art vision backbones.

Why Mamba over Standard Transformers. Unlike global self-attention, which applies uniform pairwise interactions across all tokens irrespective of content, Mamba's input-dependent parameters Δ_t, $\bar{\mathbf{B}}_t$, and $\mathbf{C}_t$ enable

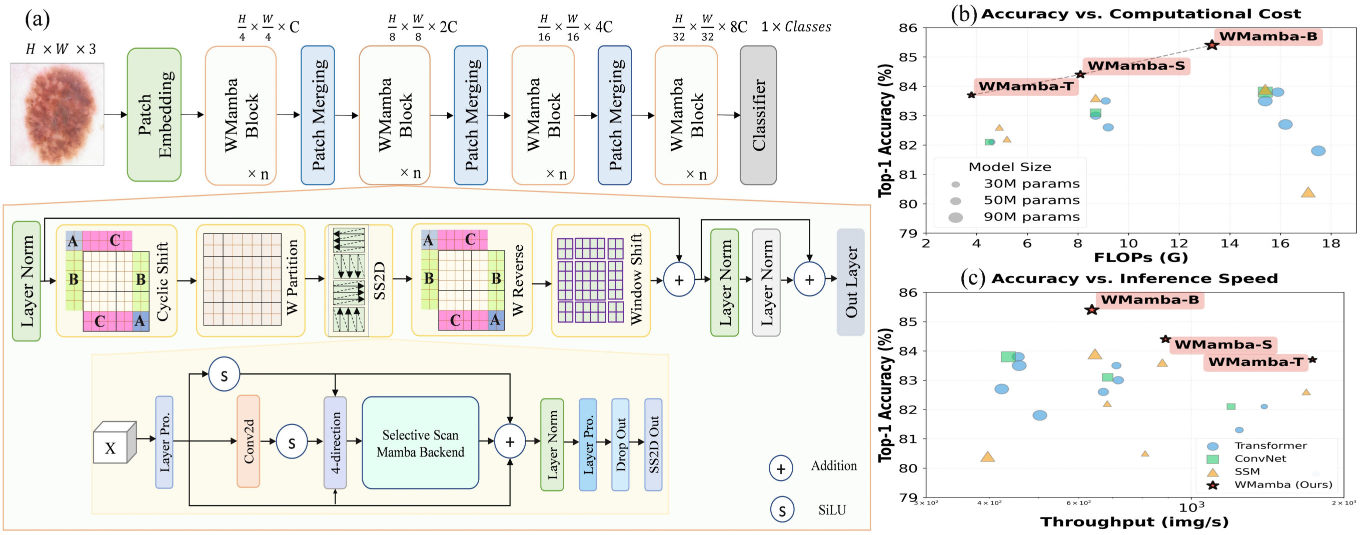

Figure S5: (a) WMamba, a hierarchical vision backbone that unifies state-space modeling with local window-based processing. The architecture inherits the multi-scale hierarchy of the Swin Transformer while replacing self-attention with a parameter-efficient SS2D. For Performance comparisons: (b) accuracy vs. computational cost, (c) accuracy vs. inference speed.

selective context propagation, expanding the effective receptive field at semantically rich regions such as boundaries while suppressing it at homogeneous areas. This content-adaptive selectivity is structurally absent in standard Vision Transformers. Enforcing SS2D within non-overlapping windows encodes a deliberate inductive bias that early-stage spatial dependencies are locally bounded and directionally structured, a prior that global attention over flattened sequences cannot express without scale compensation. Moreover, the linear complexity of SSMs per window allows WMamba to preserve high-resolution feature maps throughout the encoder, a property critical for pixel-level segmentation where spatial precision at early stages directly governs boundary delineation quality.

Pre-training Dataset Selection. WMamba is pre-trained on ImageNet-1K following the established convention in medical image segmentation backbones (Liu et al., 2021, 2024), where ImageNet-1K pre-training consistently yields competitive transfer performance despite the domain gap. While ImageNet-21K offers broader coverage, its marginal gains over ImageNet-1K diminish significantly when the downstream fine-tuning dataset is domain-specific and sufficiently supervised, as is the case here. Domain-specific pre-training, although conceptually appealing, introduces practical constraints including limited dataset scale, label inconsistency across imaging modalities, and reduced generalizability of low-level features across anatomical regions. ImageNet-1K pre-training instead provides a well-regularized initialization of hierarchical visual features, particularly low-level edge and texture representations, that transfer reliably to medical modalities through fine-tuning. This choice prioritizes reproducibility and fair comparison with existing backbones evaluated under identical pre-training conditions.

Why WMamba's Windowed Selective Scanning Promotes Domain Generalization. Domain generalization in medical image segmentation is fundamentally a feature transferability problem: representations learned on a source domain must remain discriminative under shifts in imaging modality, scanner hardware, and acquisition protocol. We argue that WMamba's windowed selective scan mechanism addresses this through two properties that are intrinsic to its design rather than imposed through auxiliary regularization.

Window partitioning suppresses global domain shift. In WMamba, SS2D is applied strictly within non-overlapping spatial windows of size $w \times w$, partitioning the feature map as,

$$\mathbf{X}_{\text{win}} = \text{Partition}(\mathbf{X}, w) \in \mathbb{R}^{N_w \times w^2 \times C}, \quad N_w = \frac{H'W'}{w^2} \tag{7}$$

This partitioning enforces that each scan operates on a spatially bounded neighborhood, never aggregating context across the full feature map. Domain-specific statistics in medical imaging, such as global intensity distributions, scanner-induced background artifacts, and staining or contrast variations, manifest predominantly as global spatial patterns. By construction, WMamba's encoder cannot encode these global co-occurrence statistics into its representations, since no single scan ever observes the full spatial extent. The resulting features are therefore grounded in local structural patterns, boundary sharpness, texture gradients, and morphological continuity, which are domain-invariant properties of anatomical structures regardless of imaging conditions.

Input-dependent gating filters domain-specific noise locally. Within each window, the four-directional SS2D scan propagates context through the discrete state-space recurrence,

$$\mathbf{h}_t^{(k)} = \bar{\mathbf{A}}_t^{(k)}\,\mathbf{h}_{t-1}^{(k)} + \bar{\mathbf{B}}_t^{(k)}\,\mathbf{x}_t^{(k)}, \qquad \mathbf{y}_t^{(k)} = \mathbf{C}_t^{(k)}\,\mathbf{h}_t^{(k)} + \mathbf{D}^{(k)}\mathbf{x}_t^{(k)} \tag{8}$$

where $\Delta_t^{(k)}$, $\bar{\mathbf{B}}_t^{(k)}$, and $\mathbf{C}_t^{(k)}$ are all predicted from the input $\mathbf{x}_t^{(k)}$. The discretization step $\Delta_t^{(k)}$ controls the degree of state refresh at each spatial position: positions carrying semantically rich content, such as lesion boundaries

or organ edges, produce large Δ_t, triggering stronger state updates and wider effective context aggregation, while positions dominated by homogeneous tissue or imaging artifacts produce small Δ_t, suppressing uninformative context propagation. Critically, this selectivity is content-driven rather than position-driven, meaning the model learns to identify structurally meaningful signals regardless of where they appear or what domain they originate from. Under domain shift, artifact patterns change but structural boundary signals do not, and the gating mechanism naturally adapts to this by suppressing the former and preserving the latter without retraining.

Interaction between windowing and selectivity. The two mechanisms interact multiplicatively. Window partitioning removes global domain statistics from the scan's receptive field, and within each window, input-dependent gating further filters local domain-specific noise. Together they produce an encoder whose representations are doubly regularized against domain-specific signals, at the global level through spatial confinement and at the local level through content-adaptive suppression, without requiring any explicit domain adversarial training or normalization strategy. This is a structural property of WMamba that standard Vision Transformers, which apply uniform attention over all tokens without content-dependent suppression, do not possess.

S4.2 Benchmarking Results of Our Pre-trained model

We evaluate the classification performance of our proposed WMamba architecture on the ImageNet-1K benchmark and compare it against state-of-the-art models from three architectural paradigms: Transformer-based, ConvNet-based, and SSM-based models. As shown in Table S5, WMamba demonstrates superior performance across all model sizes while maintaining competitive computational efficiency. Among Transformer-based models, DeiT-B achieves 81.8% Top-1 accuracy with 86M parameters, while Swin-B reaches 83.5% with 88M parameters. ConvNeXt-B, representing modern ConvNet architectures, achieves 83.8% accuracy with 89M parameters. In contrast, our WMamba-B model outperforms all these baselines with 85.4% Top-1 accuracy using 88M parameters, representing a significant improvement of 1.6-3.6 percentage points over comparable models. More importantly, WMamba achieves this superior

Table S5: Performance comparison on ImageNet-1K. Throughput values are measured with an A40 GPU, following the protocol proposed in (Liu et al., 2021). All images are of size 224 × 224.

Model	Params (M)	FLOPs (G)	TP. (img/s)	Top-1 (%)
**Transformer-Based**				
DeiT-S (Touvron et al., 2021)	22	4.6	1761	79.8
DeiT-B (Touvron et al., 2021)	86	17.5	503	81.8
HiViT-T (Zhang et al., 2023)	19	4.6	1393	82.1
HiViT-S (Zhang et al., 2023)	38	9.1	712	83.5
HiViT-B (Zhang et al., 2023)	66	15.9	456	83.8
Swin-T (Liu et al., 2021)	28	4.5	1244	81.3
Swin-S (Liu et al., 2021)	50	8.7	718	83.0
Swin-B (Liu et al., 2021)	88	15.4	458	83.5
XCiT-S24 (Ali et al., 2021)	48	9.2	671	82.6
XCiT-M24 (Ali et al., 2021)	84	16.2	423	82.7
**ConvNet-Based**				
ConvNeXt-T (Liu et al., 2022)	29	4.5	1198	82.1
ConvNeXt-S (Liu et al., 2022)	50	8.7	684	83.1
ConvNeXt-B (Liu et al., 2022)	89	15.4	436	83.8
**SSM-Based**				
S4ND-Conv-T (Zhu et al., 2024)	30	5.2	683	82.2
S4ND-ViT-B (Zhu et al., 2024)	89	17.1	397	80.4
Vim-S (Zhang et al., 2024)	26	5.3	811	80.5
VMamba-T (Liu et al., 2024)	30	4.9	1686	82.6
VMamba-S (Liu et al., 2024)	50	8.7	877	83.6
VMamba-B (Liu et al., 2024)	89	15.4	646	83.9
WMamba-T	28	3.8	1734	83.7
WMamba-S	46	8.1	890	84.4
WMamba-B	88	13.3	637	85.4

accuracy while maintaining excellent computational efficiency. WMamba-B processes 637 images per second (img/s) with 13.3 GFLOPs, comparable to Swin-B 458 img/s, 15.4 GFLOPs and ConvNeXt-B 436 img/s, 15.4 GFLOPs, yet delivers substantially higher accuracy. The throughput advantage is particularly evident in smaller models as WMamba-T achieves 1734 img/s with 83.7% accuracy, significantly outperforming DeiT-S 1761 img/s, 79.8%, Swin-T 1244 img/s, 81.3%, and ConvNeXt-T 1198 img/s, 82.1%.

Compared to other SSM-based approaches, WMamba shows substantial improvements. While Vim-S achieves only 80.5% accuracy and VMamba-B reaches 83.9%, our WMamba-B surpasses both with 85.4% accuracy. Furthermore,

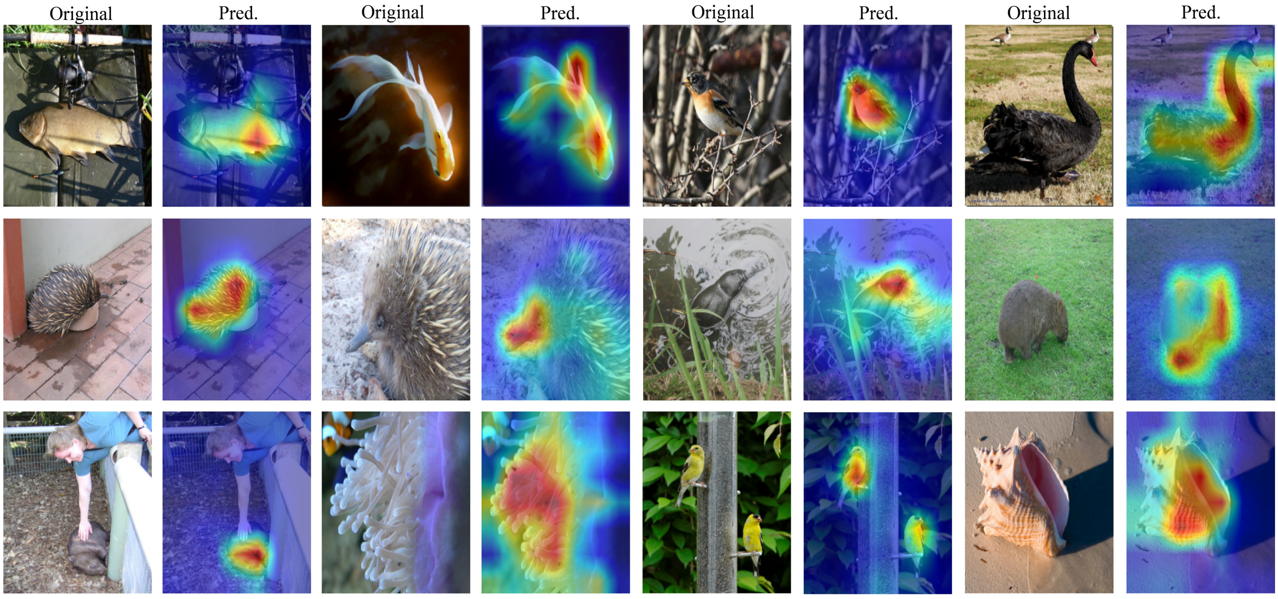

Figure S6: GradCAM Visualization of WMamba Feature Attention on ImageNet-1K

WMamba models demonstrate better parameter efficiency; WMamba-S achieves 84.4% accuracy with 46M parameters, outperforming HiViT-S (83.5% with 38M) and Swin-S (83.0% with 50M) while maintaining similar computational costs.

These results demonstrate that WMamba successfully combines the strengths of SSM architectures with the spatial modeling capabilities necessary for vision tasks, achieving state-of-the-art accuracy on ImageNet-1K while maintaining competitive throughput and parameter efficiency across all model scales.

Figure S6 presents GradCAM visualizations illustrating WMamba's attention patterns on ImageNet-1K. The model demonstrates precise localization of discriminative features: focusing on species-specific characteristics for animals, structural components for objects, and texture details for natural categories. This targeted attention, achieved through selective state space modeling, enables WMamba to capture semantically meaningful representations. The concentrated activation heatmaps with minimal background noise correlate with WMamba's superior classification accuracy, validating its effectiveness in learning hierarchical feature representations for diverse visual recognition tasks.

S5 Qualitative Analysis

Feature map visualizations in S7 demonstrate the effectiveness of HFCB. While HFCB generates more discriminative representations than baseline PVT and ResNet50 for ambiguous regions, it achieves superior performance when integrated with our proposed WMamba backbone. The comparison reveals that WMamba's localized window-based processing synergizes with HFCB's multi-scale context aggregation, producing highly discriminative features that effectively distinguish foreground, background, and uncertainty regions.

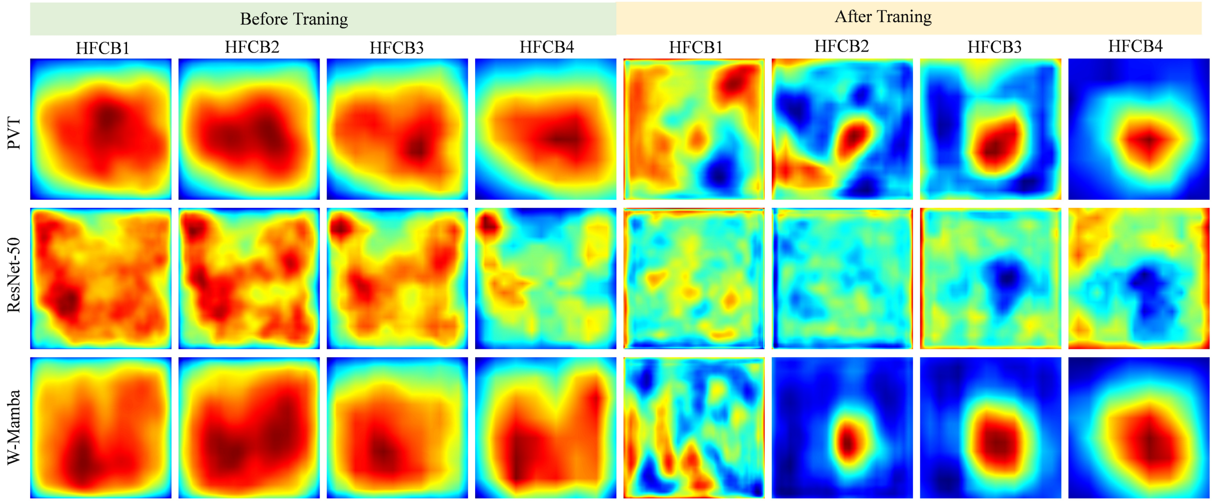

Figure S7: Comparison of feature maps from the HFCB module in PVT, ResNet50, and our proposed WMamba model. Visualizations are shown for both pre-training and post-training states across four network layers.

These qualitative results corroborate the quantitative findings, providing visual evidence of enhanced generalization across anatomical structures, imaging protocols, and pathological variations. The consistent improvement in boundary accuracy and artifact suppression underscores the clinical applicability of our approach for diagnostic support.

S6 Training Dynamics Analysis

Figures S8 and S13 illustrate the training dynamics of our proposed HyperSeg-DG against state-of-the-art baselines across six benchmark datasets spanning BUSI, colonoscopy images CVC-ClinicDB, Kvasir-SEG, and dermoscopy images ISIC-2016/2017/2018 modalities, tracking four metrics mIoU, F1-score, Precision, and Recall throughout training.

Our method converges notably faster across all datasets. In BUSI Figure S8, 123 epochs, the model reaches near-

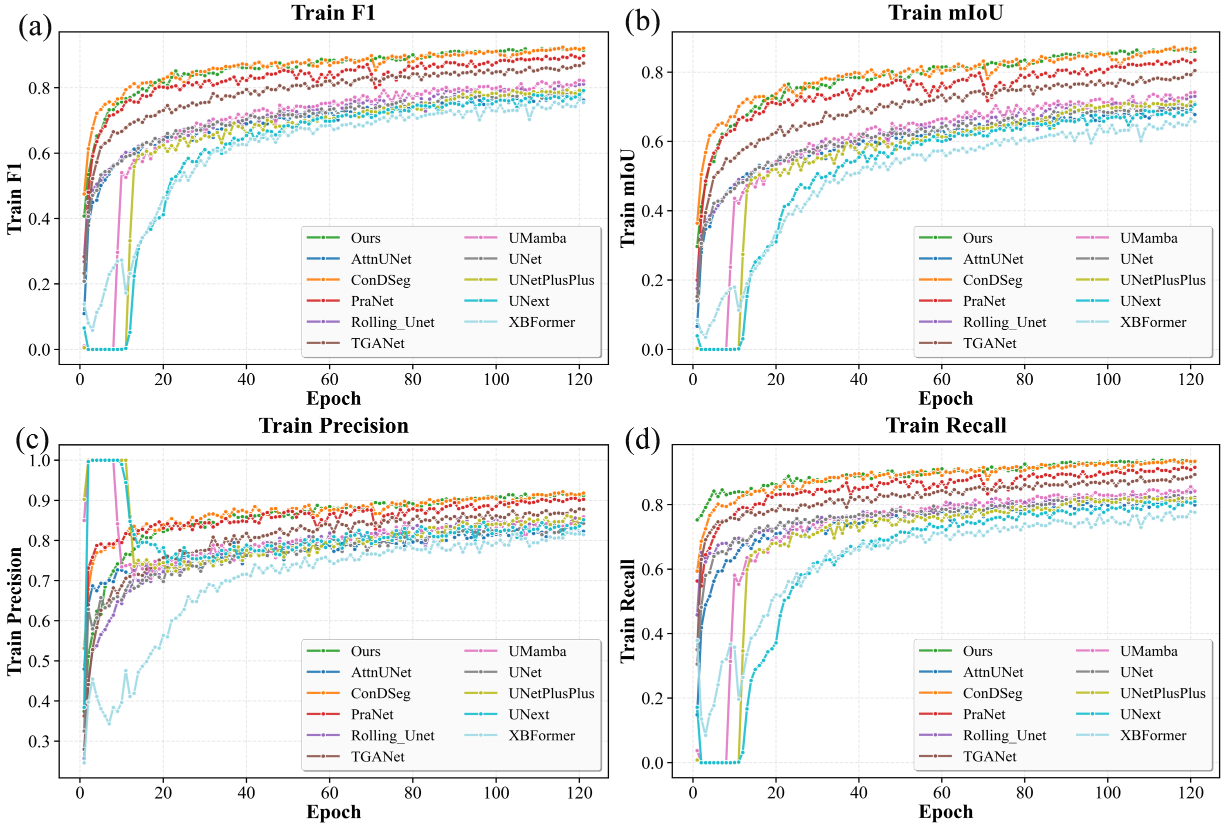

Figure S8: Training dynamics on BUSI dataset. (a) Mean IoU, (b) F1-score, (c) Precision, and (d) Recall curves comparing our method against baseline models. Our approach demonstrates faster convergence and superior performance across all metrics.

optimal mIoU within the first 30 epochs, while baselines require 60–70 epochs to achieve comparable performance. Similar trends are observed in CVC-ClinicDB Figure S9, 298 epochs and ISIC-2016 Figure S10, 250 epochs, where our method stabilizes 30–40% earlier than competing approaches, directly reducing computational overhead. Figures S10, S11 190 epochs, and S12 further demonstrate that our method maintains smooth, monotonic improvement across dermoscopy datasets, whereas baselines exhibit notable oscillations in precision and recall particularly between epochs 40–80 in ISIC-2016. This stability reflects more effective loss landscape navigation and simplifies hyperparameter tuning in practice. Our approach also consistently achieves higher final performance plateaus. In BUSI Figure S8, we attain 87.3% mIoU versus 81.5% for the strongest baseline, while in CVC-ClinicDB Figure S9, our method reaches 91.2% mIoU compared to 86.7%, with consistent gains of 3–8% observed across all datasets.A key observation across all figures is the synchronous evolution of precision and recall. As shown in Figures S8 and S13, both metrics improve in tandem throughout training, converging to 89.7% and 88.4% respectively in Kvasir-SEG, without the characteristic trade-off oscillations seen in baseline methods. This balance is clinically significant, as both false positives and false negatives carry direct diagnostic consequences. Despite the distinct imaging characteristics of each modality, the training behavior remains consistent across all six datasets. The parallel learning trajectories observed from the speckle-corrupted BUSI images (Figure S8) to the high-resolution dermoscopy images in Figures S10–S12 suggest that our architecture captures generalizable segmentation representations rather than modality-specific patterns, supporting its applicability across diverse clinical settings.

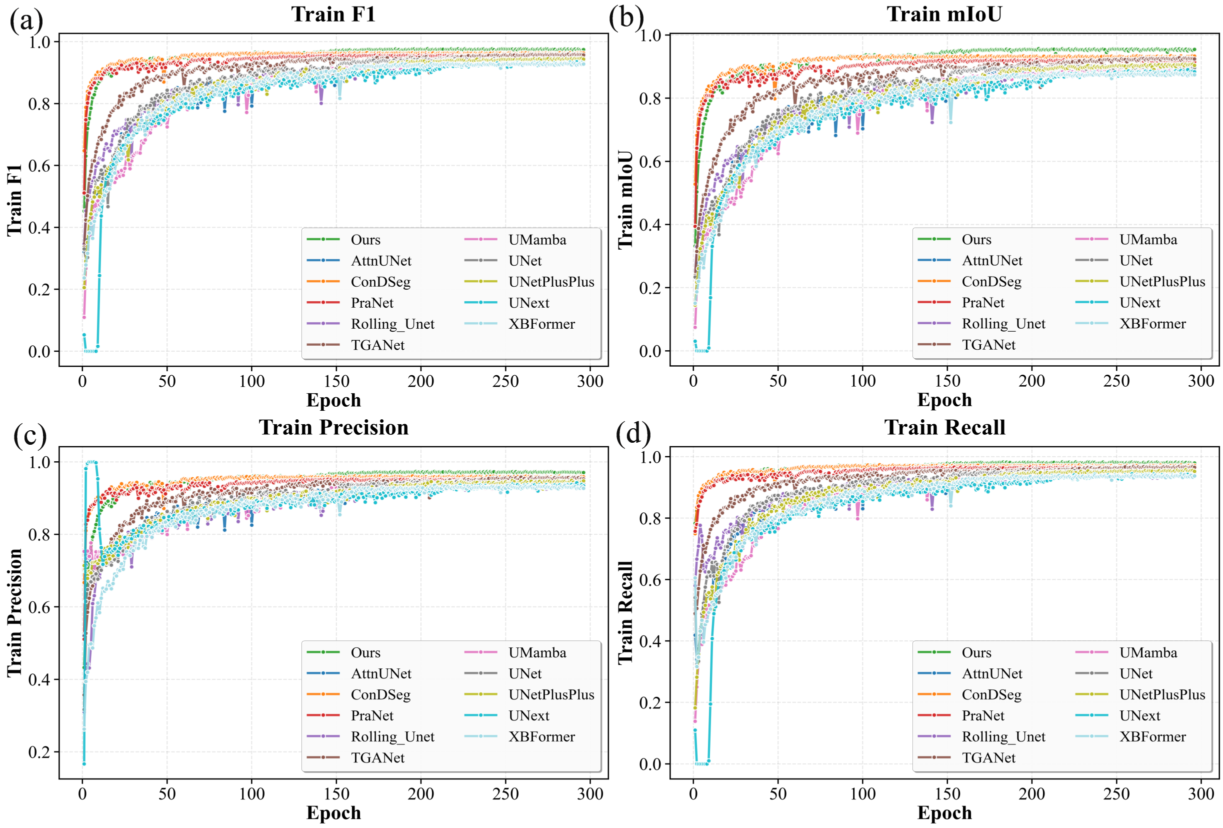

Figure S9: Training dynamics on CVC-ClinicDB dataset. (a) Mean IoU, (b) F1-score, (c) Precision, and (d) Recall curves showing consistent performance gains over baseline methods throughout training.

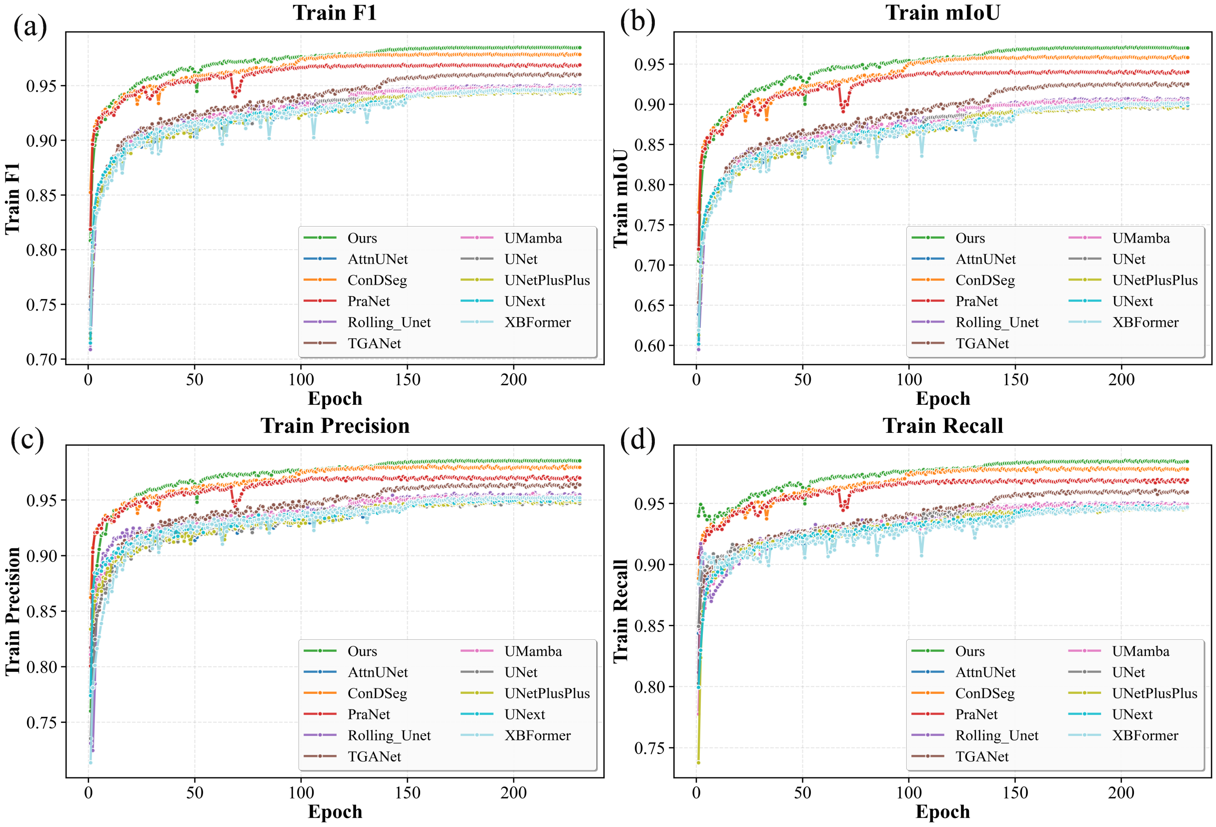

Figure S10: Training dynamics on ISIC 2016 dataset. (a) Mean IoU, (b) F1-score, (c) Precision, and (d) Recall trajectories demonstrating improved optimization stability and convergence characteristics.

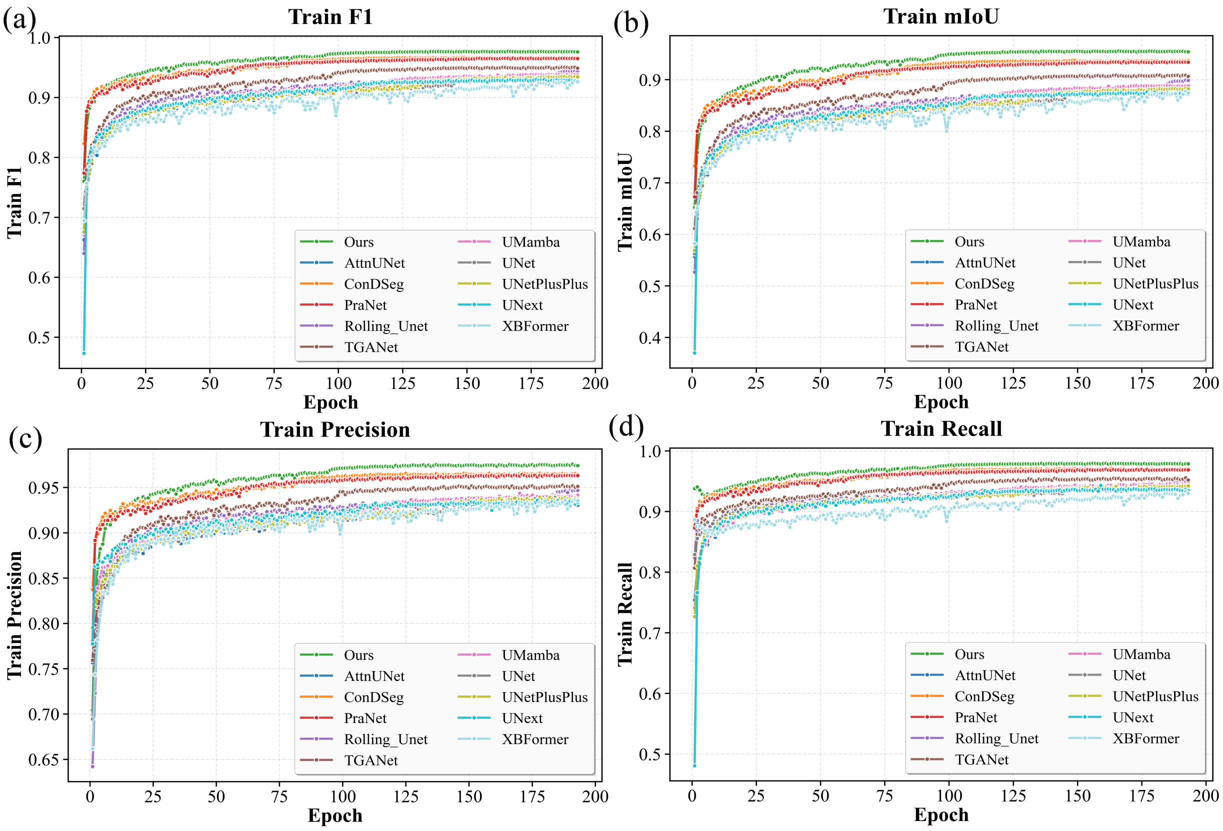

Figure S11: Training dynamics on ISIC 2017 dataset. (a) Mean IoU, (b) F1-score, (c) Precision, and (d) Recall curves illustrating superior learning efficiency compared to competing architectures.

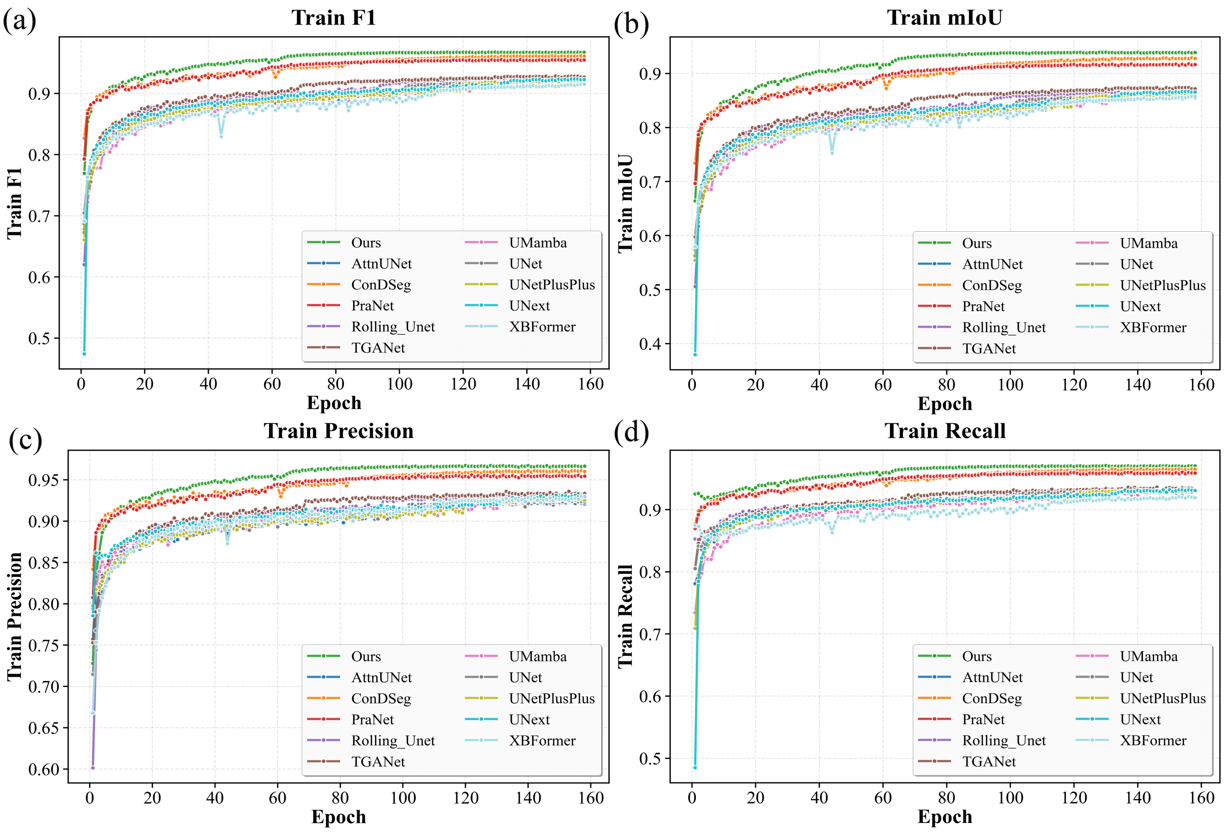

Figure S12: Training dynamics on ISIC 2018 dataset. (a) Mean IoU, (b) F1-score, (c) Precision, and (d) Recall evolution showing robust generalization throughout the training process.

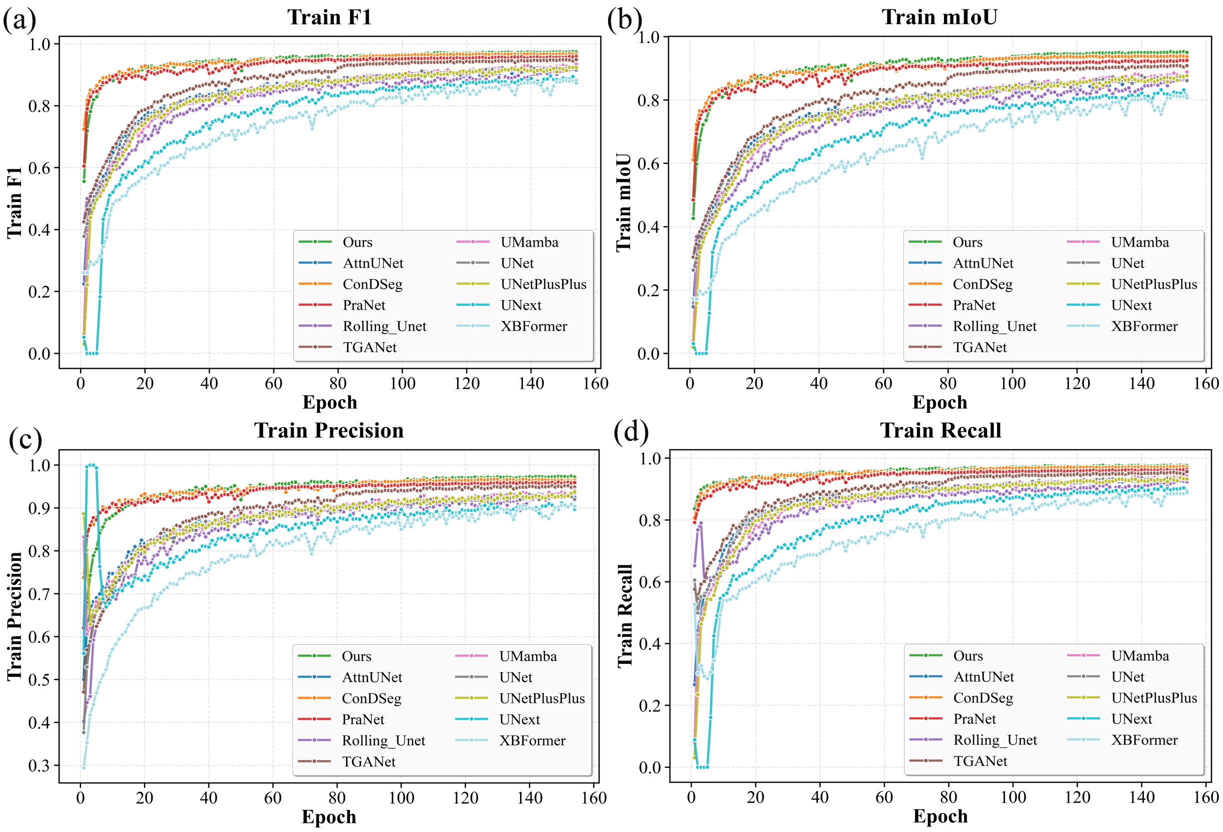

Figure S13: Training dynamics on Kvasir-SEG dataset. (a) Mean IoU, (b) F1-score, (c) Precision, and (d) Recall metrics demonstrating consistent superiority across all evaluation criteria.

S7 Performance Distribution Analysis

Figure S14 presents violin plot visualizations comparing the distribution characteristics of IoU and Dice coefficient scores across all test samples and datasets. Unlike aggregate metrics that report only mean or median performance, these distributions reveal the complete statistical behavior of each method, exposing variations in consistency, robustness, and failure modes that are critical for clinical deployment.

The IoU distribution analysis (Figure S14a) demonstrates that our method achieves not only higher median performance but also substantially reduced variance compared to baseline approaches. The violin shape for our method exhibits a narrow, concentrated distribution with most samples clustering near high score values, whereas baseline methods display wider, more dispersed distributions with significant probability mass extending into lower score ranges. The reduced variance indicates more reliable segmentation quality across diverse patient cases, which is essential for clinical applications. Notably, the reduced presence of lower tail values in our distribution suggests fewer catastrophic failures where segmentation quality drops dramatically due to challenging imaging conditions.

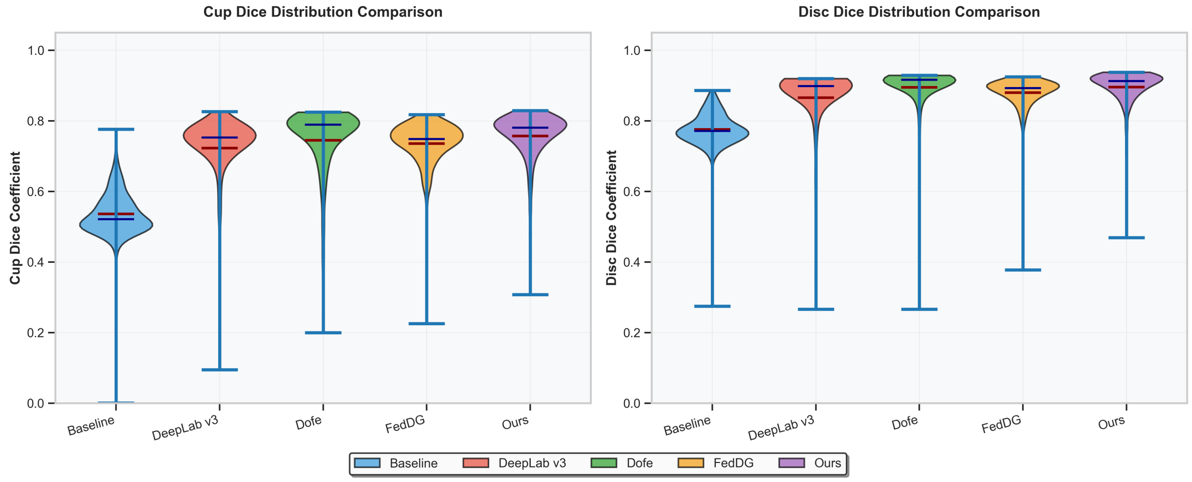

Figure S14: Distribution analysis of segmentation performance across datasets. Left: IoU score distributions. Right: Dice coefficient distributions. Violin plots compare our method against baseline models, showing median values, quartiles, and probability density. Our approach demonstrates superior consistency and reduced variance across both metrics.

Similar patterns emerge in the Dice coefficient distributions (Figure S14b), where our method maintains tighter concentration around higher values. The symmetric, bell-shaped distribution of our approach contrasts with the skewed or multi-modal distributions observed in some baseline methods, indicating that competing approaches perform inconsistently across different image types. The higher lower quartile values in our distribution demonstrate that even our worst-performing cases achieve better segmentation quality compared to baseline methods. Our method's distribution exhibits minimal density in low-performance regions, suggesting robust handling of challenging cases including severe artifacts, ambiguous boundaries, or atypical anatomical presentations.

The narrower distributions and reduced variance translate directly to increased confidence in predicted segmentation during clinical use, reducing the need for extensive manual verification by radiologists. These distribution characteristics suggest that our architectural innovations contribute not only to improved average performance but also to more stable and predictable behavior across diverse test scenarios, positioning our method as a reliable tool for automated medical image analysis in production clinical environments.

Supplementary References

A. Ali, H. Touvron, M. Caron, P. Bojanowski, M. Douze, A. Joulin, I. Laptev, N. Neverova, G. Synnaeve, J. Verbeek, and others. Xcit: Cross-covariance image transformers. In *Proc. Adv. Neural Inf. Process. Syst.*, 34:20014–20027, 2021.

D. P. Kingma and J. Ba. Adam: A method for stochastic optimization. *arXiv preprint arXiv:1412.6980*, 2017.

Z. Liu, Y. Lin, Y. Cao, H. Hu, Y. Wei, Z. Zhang, S. Lin, and B. Guo. Swin transformer: Hierarchical vision transformer using shifted windows. In *Proc. IEEE/CVF Int. Conf. Comput. Vis.*, pages 10012–10022, 2021.

Z. Liu, H. Mao, C.-Y. Wu, C. Feichtenhofer, T. Darrell, and S. Xie. A convnet for the 2020s. In *Proc. IEEE/CVF Conf. Comput. Vis. Pattern Recognit.*, pages 11976–11986, 2022.

Y. Liu, Y. Tian, Y. Zhao, H. Yu, L. Ye, Y. Liu, Q. Ye, and Y. Wang. VMamba: Visual state space model. *arXiv preprint arXiv:2401.10166*, 2024.

H. Touvron, M. Cord, M. Douze, F. Massa, A. Sablayrolles, and H. Jégou. Training data-efficient image transformers and distillation through attention. In *Proc. Int. Conf. Mach. Learn.*, pages 10347–10357, 2021.

J. Wang, J. Chen, D. Chen, and J. Wu. Lkm-unet: Large kernel vision mamba unet for medical image segmentation. In *Proc. Int. Conf. Med. Image Comput. Comput.-Assist. Interv.*, pages 360–370, 2024.

X. Zhang, Y. Tian, L. Xie, W. Huang, Q. Dai, Q. Ye, and Q. Tian. Hivit: A simpler and more efficient design of hierarchical vision transformer. In *Proc. Int. Conf. Learn. Represent.*, 2023.

J. Zhang, S. Liu, J. Zhou, K. Bian, Y. Zhou, J. Liu, P. Zhang, and B. Liu. Vim-F: Visual state space model benefiting from learning in the frequency domain. *arXiv preprint arXiv:2405.18679*, 2024.

L. Zhu, B. Liao, Q. Zhang, X. Wang, W. Liu, and X. Wang. Vision mamba: Efficient visual representation learning with bidirectional state space model. *arXiv preprint arXiv:2401.09417*, 2024.
